# Soft crystalline properties of 2D frameworks constructed from lithium ions and dinitriles

**DOI:** 10.1039/d5sc06222e

**Published:** 2025-12-16

**Authors:** Taichi Nishiguchi, Kotoha Kageyama, Takuya Kurihara, Nanae Shimanaka, Shun Tokuda, Shuto Tsuda, Nattapol Ma, Satoshi Horike

**Affiliations:** a Department of Chemistry, Graduate School of Science, Kyoto University Kitashirakawa-Oiwakecho, Sakyo-ku Kyoto 606-8502 Japan horike.satoshi.3r@kyoto-u.ac.jp; b Department of Synthetic Chemistry and Biological Chemistry, Graduate School of Engineering, Kyoto University Katsura, Nishikyo-ku Kyoto 615-8510 Japan; c Division of Material Chemistry, Graduate School of Natural Science and Technology, Kanazawa University Kakuma-Machi Kanazawa Ishikawa 920-1192 Japan; d Institute for Integrated Cell-Material Sciences, Institute for Advanced Study, Kyoto University Yoshida-Honmachi, Sakyo-ku Kyoto 606-8501 Japan; e Department of Materials Science and Engineering, School of Molecular Science and Engineering, Vidyasirimedhi Institute of Science and Technology Rayong 21210 Thailand

## Abstract

We constructed two-dimensional (2D) molecular frameworks composed of lithium ions (Li^+^) and dinitrile aliphatic ligands to explore their mechanical and thermal properties. Calorimetry, X-ray diffraction, density functional theory calculations, alternating current impedance, and solid-state nuclear magnetic resonance evidenced behaviours and properties originating from the weakly linked 2D system. We found low melting temperatures (<100 °C), high mechanical deformability, large positive and negative thermal expansion, and metal ion diffusion. These features were uniquely observed in the integration of Li^+^ and dinitriles into extended molecular structures.

## Abstract 2

We synthesised four crystalline frameworks from lithium ions and aliphatic dinitrile molecules. Properties based on the weak metal–ligand interactions were studied based on the understanding of well-defined crystal structures.

## Graphical abstract

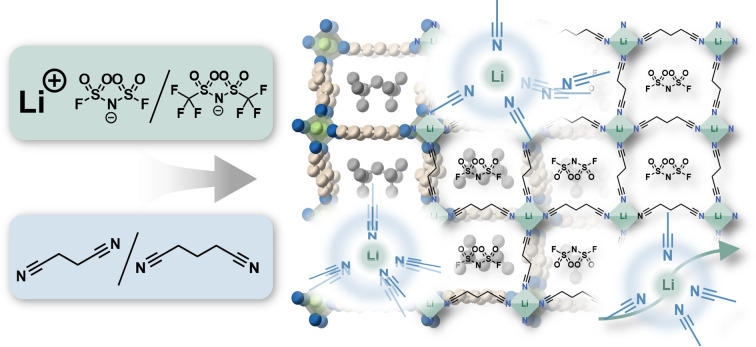

## Introduction

Coordination polymers (CPs) and metal–organic frameworks (MOFs) are molecular frameworks featuring well-defined, periodic structures. Conventional design and synthesis of CP/MOFs have focused on the robustness and stability of frameworks based on stronger coordination bonds by applying high-valent transition metal ions such as oxophilic Zr^4+^ and Cr^3+^ and rigid ligands.^1,2^ On the other hand, frameworks based on weaker metal–ligand interactions have been relatively unexplored, despite their potential to exhibit distinct dynamic and thermomechanical behaviours.

In recent studies, synthetic approaches to incorporate weak metal–ligand interactions have been adopted to create CP/MOF crystals with phase changes, including melting behaviours.^3–7^ Controlling the bond strength (enthalpic factor) and the mobility of molecular components (entropic factor) reduces the melting temperature (*T*_m_) to a value lower than the decomposition temperature, yielding a stable liquid state.^8,9^ In other words, CP/MOFs constructed from metal ions and bridging ligands with weak interactions would represent not only the chance to explore the melting phenomena but also their unique physical (especially, thermal) properties in the crystalline state; however, many of them have been overlooked.

Here, we report molecular frameworks constructed from lithium ions (Li^+^) and dinitriles. We selected Li^+^ as nodes and dinitrile aliphatic linkers to construct crystalline frameworks, utilising their non-directional bonding and the inherent flexibility of the linker molecules.^10–13^ A solvent-free melt-cooling reaction was employed instead of a self-assembling process in solutions to incorporate these components into single crystalline phases. The crystalline products are two-dimensional (2D) structures and exhibit low *T*_m_ ranging from 59 to 90 °C, below those typically reported for CP/MOFs. They exhibited Young's moduli below 18 GPa, and one compound showed large positive and negative thermal expansion, with the absolute value of the coefficient exceeding 100 × 10^−6^ K^−1^. The weak interactions between the metal and ligands facilitate dissociation of the coordination interactions, resulting in the diffusion of the metal ions in the crystalline phase.

## Results and discussion

### Crystal structures

**Fig. 1:**
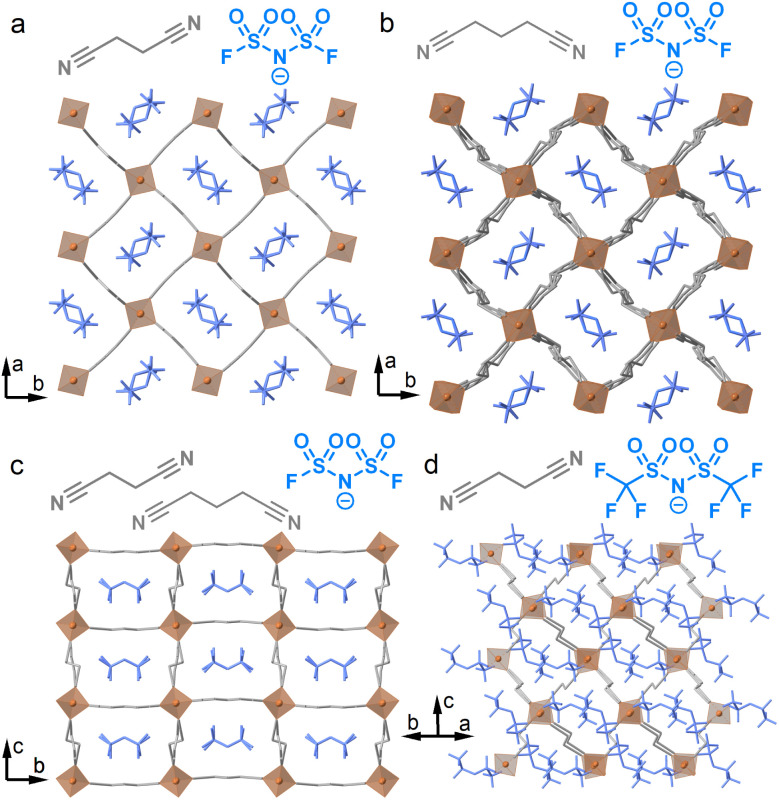
Schematic representations of dinitriles and anions, and crystal structures of (a) Li(FSI)(SN)_2_ (1) at −100 °C, (b) Li(FSI)(GN)_2_ (2) at 0 °C, (c) Li(FSI)(SN)(GN) (3) at 0 °C, and (d) Li(TFSI)(SN)_1.5_ (4) at −175 °C. SN/GN: grey, Li^+^: orange, and FSI^−^/TFSI^−^: blue. H atoms are omitted for clarity.

Four Li-based frameworks, Li(FSI)(SN)_2_ (1), Li(FSI)(GN)_2_ (2), Li(FSI)(SN)(GN) (3), and Li(TFSI)(SN)_1.5_ (4) (FSI^−^ = bis(fluorosulfonyl)imide, TFSI^−^ = bis(trifluoromethanesulfonyl)imide, SN = succinonitrile, and GN = glutaronitrile) were synthesised (see the SI for the detailed synthetic methods).^10,14–16^ A solvent-free melt-cooling reaction was performed under an Ar atmosphere to effectively trap the metastable, weak interactions in the crystalline states. This reaction is necessary because no crystals were formed using conventional solvothermal reactions with ethanol, acetonitrile, or tetrahydrofuran. In Fig. 1 and S1, we reveal the structures through single crystal X-ray diffraction (SC-XRD) analyses of 2, 3, and 4, along with the previously reported structure of 1. All obtained crystals consist of tetrahedral Li^+^ and dinitrile linkers. 2, 3, and 4 feature 2D sheet structures. Compounds 2 and 3 consist of frameworks with tetracoordinated Li^+^ and SN/GN linkers, containing FSI within them. In contrast, 4 has two inequivalent Li^+^ sites; one site is coordinated with four SN, while the other has two SN and two TFSI^−^. The TFSI^−^ anions cap the sheets to form a 2D extended structure. The 2-nm sheets exhibit a sql topology, as determined by TOPOS Pro,^17^ with a two-fold inclined interpenetration. Mixtures of Li(BF_4_) and SN, and Li(PF_6_) and SN, at the molar ratio of 1/2 formed solids with unclear melting behaviours, suggesting the formation of impurity phases. These results indicate the importance of FSI^−^ and TFSI^−^ for the formation of pure single phases.

### Thermal behaviours

**Fig. 2:**
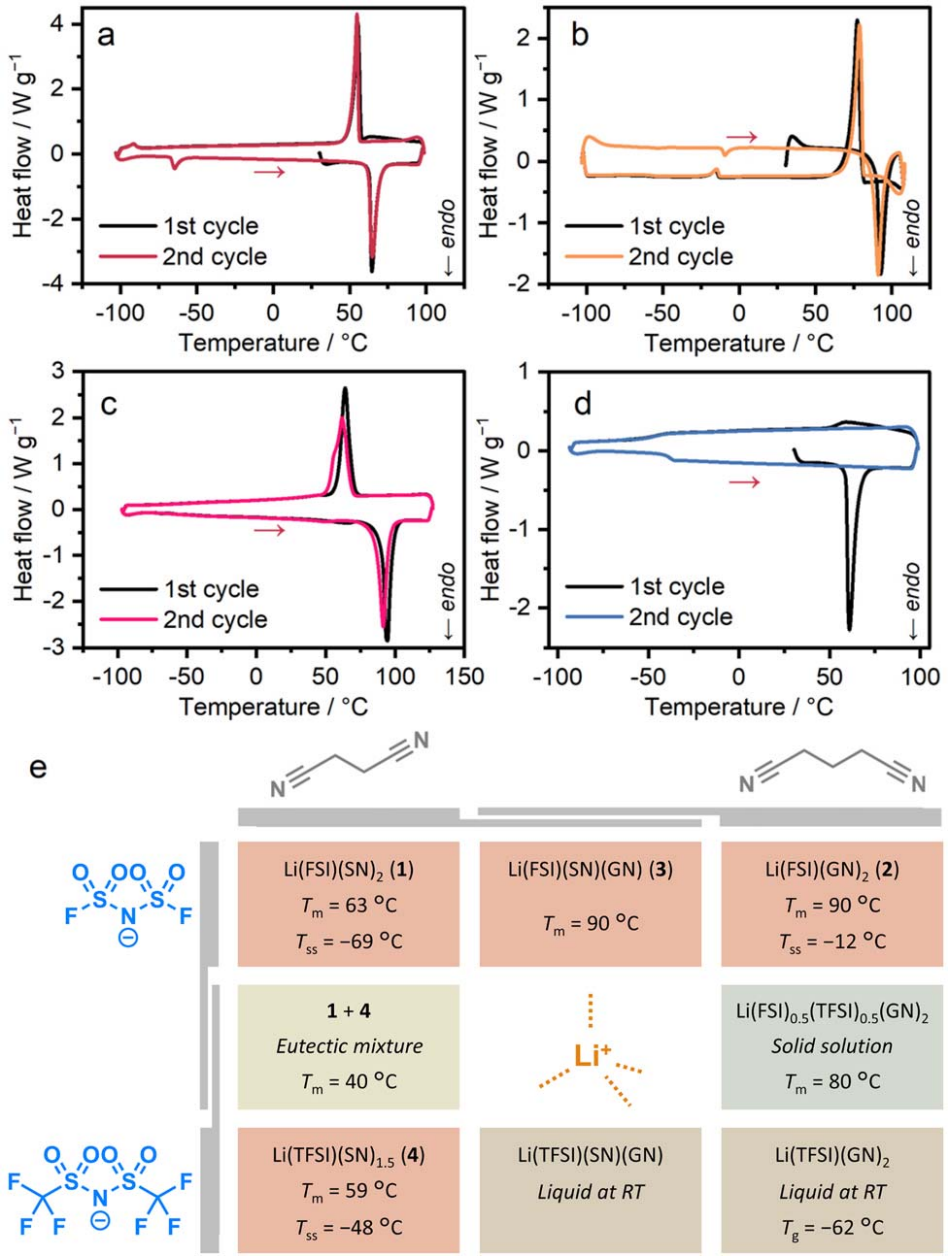
DSC profiles of (a) 1, (b) 2, (c) 3, and (d) 4. Red arrows indicate heating processes. (e) Summary of thermal and phase behaviours.

Thermogravimetric analysis (TGA) determined the decomposition temperatures of 1–4 to be 109, 140, 139, and 139 °C (Fig. S2), forming a stable liquid state over a temperature window of 50 °C. In Fig. 2, differential scanning calorimetry (DSC) revealed melting peaks for 1–4 at 63, 90, 90, and 59 °C (Fig. 2a–d). *T*_m_ of 1 aligns with the reported value, suggesting high phase purity.^10^ Variable-temperature (VT) powder X-ray diffraction (PXRD) of powder samples, matching the patterns simulated from SC-XRD structures, confirmed the complete transformation during melting (Fig. S3). We synthesised Li(TFSI)(bpe)_2_ (bpe = 1,2-bis(4-pyridyl)ethane) to elucidate the role of the ligand in reducing *T*_m_ (Fig. S4 and Table S1). It showed a *T*_m_ of 219 °C in DSC, without significant weight loss in TGA (Fig. S5), a temperature comparable to reported TFSI^−^- and pyridine-based melting CPs and analogues (168–245 °C).^18–21^ This indicates that the origin of the low *T*_m_ of 1–4 arises from the weak interaction between Li^+^ and nitrile groups. In the DSC of 4, a glass transition was observed at −48 °C (*T*_g_) in the second heating process. This indicates the low diffusivity of the components due to the bulky TFSI^−^ and a high Li^+^/linker ratio, suppressing crystallisation to form a glassy state.^22^ We performed DSC at different ramping rates (Fig. S6). While ramping rates of 3 K min^−1^ or higher observed no crystallisation, the heating process at 2 K min^−1^ resulted in crystallisation. This indicates that ramping rates above 2 K min^−1^ are required for the formation of a stable glassy/liquid phase of 4.

To gain an understanding of low-melting behaviours, we studied the thermodynamic parameters. The melting enthalpy (Δ*H*_fus_) and melting entropy (Δ*S*_fus_) for 1, 2, 3, and 4 are presented in Table 1. The lower Δ*H*_fus_ for 2 compared to 1 results from the increased cation–anion distances, which reduce the electrostatic stability of 2.^8,23^ According to the equation *T*_m_ = Δ*H*_fus_/Δ*S*_fus_, a higher Δ*H*_fus_ corresponds to a higher *T*_m_. However, 2 exhibits a higher *T*_m_ than 1 despite its lower Δ*H*_fus_. This can be attributed to the significantly lower Δ*S*_fus_ of 2, highlighting the important role of entropy, which has been discussed in relation to ionic liquids and CPs.^20,24^ Among the low-melting MOFs, entropy also plays a significant role in their melting behaviours. The lower Δ*S*_fus_ of 2, despite the longer alkyl chain, indicates a higher degree of freedom, or more ‘liquid-like’ dynamics of GN in 2. The high Δ*H*_fus_ and Δ*S*_fus_ of 3 originate from the more densely packed structure, supported by the density (Table S1). The lower Δ*H*_fus_ and Δ*S*_fus_ of 4 are explained by the lower symmetry of the crystal and fewer aliphatic components in the composition.

**Table 1:** Thermodynamic parameters of 1–4 and 1_0.5_4_0.5_

Sample	*T*_m_/°C	Δ*H*_fus_/kJ mol^−1^	Δ*S*_fus_/J K^−1^ mol^−1^	*T*_ss_/°C	Δ*H*_ss_/kJ mol^−1^	Δ*S*_ss_/J K^−1^ mol^−1^	*T*_g_/°C
1	63	34	100	−69	3.8	18.5	—
2	90	29	80	−12	1.3	5.0	—
3	90	39	107	—	—	—	—
4	59	27	81	—	—	—	−48
1_0.5_4_0.5_	40	30	97	—	—	—	—

In addition to melting, small sharp peaks were observed in DSC of 1 and 2 at −69 and −12 °C (*T*_ss_). The solid–solid transition (SST) entropies (Δ*S*_ss_) of 1 and 2 were 18.5 and 5.0 J K^−1^ mol^−1^. They are equal to 2.2 *R* and 0.6 *R*, where *R* is the universal gas constant. These results indicate an increase in the number of states (*W*) by 9.3 and 1.8 times, as derived from Boltzmann's equation, *S*=*R* In(*W*).^25^ The *W* of 1.8 for 2 is almost equal to 2, and this is consistent with the disordering of GN into two possible conformations at a higher temperature than *T*_ss_. The *W* of 9.3 for 1 suggests the disordering of both SN and FSI^−^ in the SST.

### Phase behaviours of mixtures and analogues

We investigated the mixture of the crystals and analogues to further understand the phase behaviours of the systems (Fig. 2e). We applied physical mixing for 1 and 2 with equimolar amounts. The PXRD pattern after five-minute hand grinding in a mortar matched that of 3, without diffraction peaks of 1 or 2 (Fig. S7). The pattern was fitted with the space group of *Pbcm*, supporting the formation of 3 (Fig. S8 and Table S2). This indicates the formation of 3, suggesting high diffusivity of the components and formability of the crystalline state of 3.

For the mixture of 1 and 4, we studied 1*_x_*4_1−_*_x_* (*x* = 0.25, 0.5, 0.75), prepared by melt-cooling processes of the mixtures with a molar ratio of *x*/(1 − *x*) on a Li^+^ basis. PXRD patterns after the melt-cooling process confirmed the co-presence of 1 and 4 crystal phases in all the mixtures without forming any new phases (Fig. S9). While 1_0.25_4_0.75_ and 1_0.75_4_0.25_ showed two melting peaks in DSC, 1_0.5_4_0.5_ exhibited a single melting peak at 40 °C (Fig. S10). These indicate the formation of a eutectic mixture with a *T*_m_ of 40 °C.^26,27^ The Δ*H*_fus_ and Δ*S*_fus_ were analysed to elucidate the thermodynamic origin of the reduction of *T*_m_ (Table 1). The Δ*H*_fus_ value of 30 kJ mol^−1^ is comparable to the average of 1 and 4, suggesting the absence of enthalpic stabilisation by mixing the crystals. Δ*S*_fus_ was calculated as Δ*H*_fus_/*T*_m_ = 97 J K^−1^ mol^−1^. This value is larger than the average of 1 and 4 ([101 + 80]/2 = 91) by 6 J K^−1^ mol^−1^, indicating the entropic contributions to the reduction of *T*_m_. The value of 6 J K^−1^ mol^−1^ is close to *R* ln(2), and this is interpreted as the term of mixing entropy (Δ*S*_mix_) in the liquid state, which is given by the equation for ideal solutions as Δ*S*_mix_ = − *R* [*x* ln(*x*) + (1 − *x*) ln (1 − *x*)]. These results highlight the pure and significant effect of mixing entropy on the minimisation of the *T*_m_ of the mixture.

The mixture of Li(FSI), Li(TFSI), and GN at the molar ratio of 1 : 1 : 4 formed a colourless crystalline powder. DSC of the product showed a sharp melting peak at 80 °C (Fig. S11), suggesting the formation of a pure crystalline phase. The PXRD pattern showed peaks at positions comparable to those of 2 (Fig. S12), proving the formation of a solid solution state with randomised anion positions with the formula of Li(FSI)_0.5_(TFSI)_0.5_(GN)_2_. We performed fitting of the PXRD pattern at 30 °C (Fig. S13). The extracted lattice constants are 0.1% larger than those of 2 (Tables S3 and S5), suggesting the expansion of the unit cell by including TFSI^−^ inside the frameworks.

We also attempted the synthesis of Li(TFSI)–GN and Li(TFSI)–SN–GN frameworks with the corresponding formula of Li(TFSI)(GN)_2_ and Li(TFSI)(SN)(GN). Each mixture of the precursors gave liquid states that remained uncrystallised over three years, forming a stable liquid state at room temperature. The DSC of Li(TFSI)(GN)_2_ observed a glass transition at −62 °C (Fig. S14), a temperature lower than the *T*_g_ of 4 by 14 °C, suggesting a possible *T*_m_ of 38 °C, assuming the *T*_g_*T*_m_ ratio based on that of 4 (0.68, for the absolute temperatures). This suggests a *T*_m_ close to RT, preventing crystal formation and resulting in the room temperature liquid phase.

### Mechanical properties

**Fig. 3:**
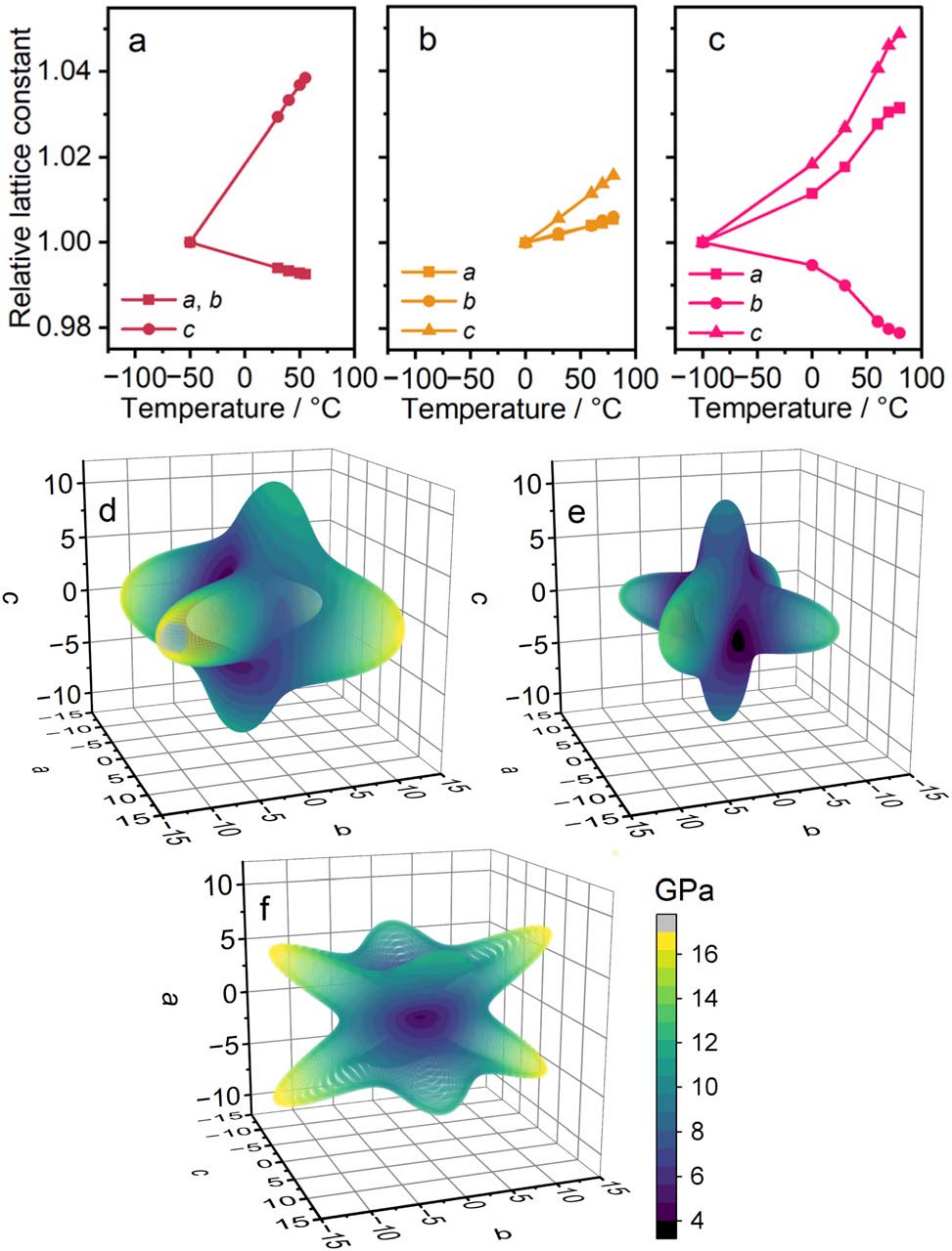
Mechanical properties of 1–3. Relative lattice constants of (a) 1, (b) 2, and (c) 3. Computed spatial dependence of Young's moduli of (d) 1, (e) 2, and (f) 3.

To investigate the mechanical properties associated with the weak metal–linker interaction, thermal expansivity was calculated from VT-PXRD patterns (Fig. S15–S17 and Tables S4–S6). Fig. 3a–c display the relative lattice constants of 1–3 as functions of temperature. For 1 and 2, temperatures above each *T*_ss_ were taken into account. The coefficient of thermal expansion (CTE) was −69 × 10^−6^ and +370 × 10^−6^ K^−1^ for the *a*/*b*- and *c*-axes of 1, and +69 × 10^−6^, +76 × 10^−6^ and +200 × 10^−6^ K^−1^ for the *a-*, *b*-, and *c*-axes of 2. The CTE is substantial compared to reported CP/MOFs and is comparable to that of organic polymers.^28^ The negative thermal expansion (NTE) in 1 is understood as a result of expansion along the *c*-axis and flexible deformation of the 3D network. For 3, the CTE was +270, +180, and −110 × 10^−6^ K^−1^ for the *a*-, *b*-, and *c*-axes, across the temperature range of −100–80 °C. Both positive and negative thermal expansions are considered ‘colossal’, reaching the CTE as large as ±100 × 10^−6^ K^−1^, and significant anisotropic expansion was observed.^29,30^ The NTE in 3 indicates the contraction of the Li–GN–Li linkage, suggesting that the higher deformability of the longer aliphatic chain contributes to the softer mechanical properties.

The mechanical properties of 1–3 were computed. The elastic tensors are calculated using the density functional theory (DFT) method (Fig. S18 and S19).^31,32^Fig. 3d–f show the spatial dependence of Young's moduli. The anisotropies in the *ab* plane of 1 and 2 are attributed to the low-symmetric initial structures considered in DFT calculations.^33,34^ The calculated Young's moduli range 4.3–17.7 (1), 3.3–13.7 (2), and 0.3–16.8 GPa (3). These values are smaller compared with reported CP/MOFs, such as Zr_6_(OH)_4_O_4_(1,4-benzenedicarboxylate)_6_ (UiO-66, 37.1–46.3 GPa) or Zn_3_O(1,4-benzenedicarboxylate)_2,_ (MOF-5, 9.5–19.7 GPa), suggesting the effect of the weak metal–linker interaction on stiffness.^35,36^1 and 2 are stiff along the Li–SN/GN–Li linkage. The smaller Young's modulus of 2 compared to 1 suggests the softness of the longer aliphatic chain of GN relative to SN. Despite similarities in the crystal structures, 3 exhibits a different shape of spatial dependence. The low Young's modulus along the *a*-axis of 3 indicates the deformability of the zig-zag structure in the Li–SN–Li linkage.

### Diffusion behaviours

**Fig. 4:**
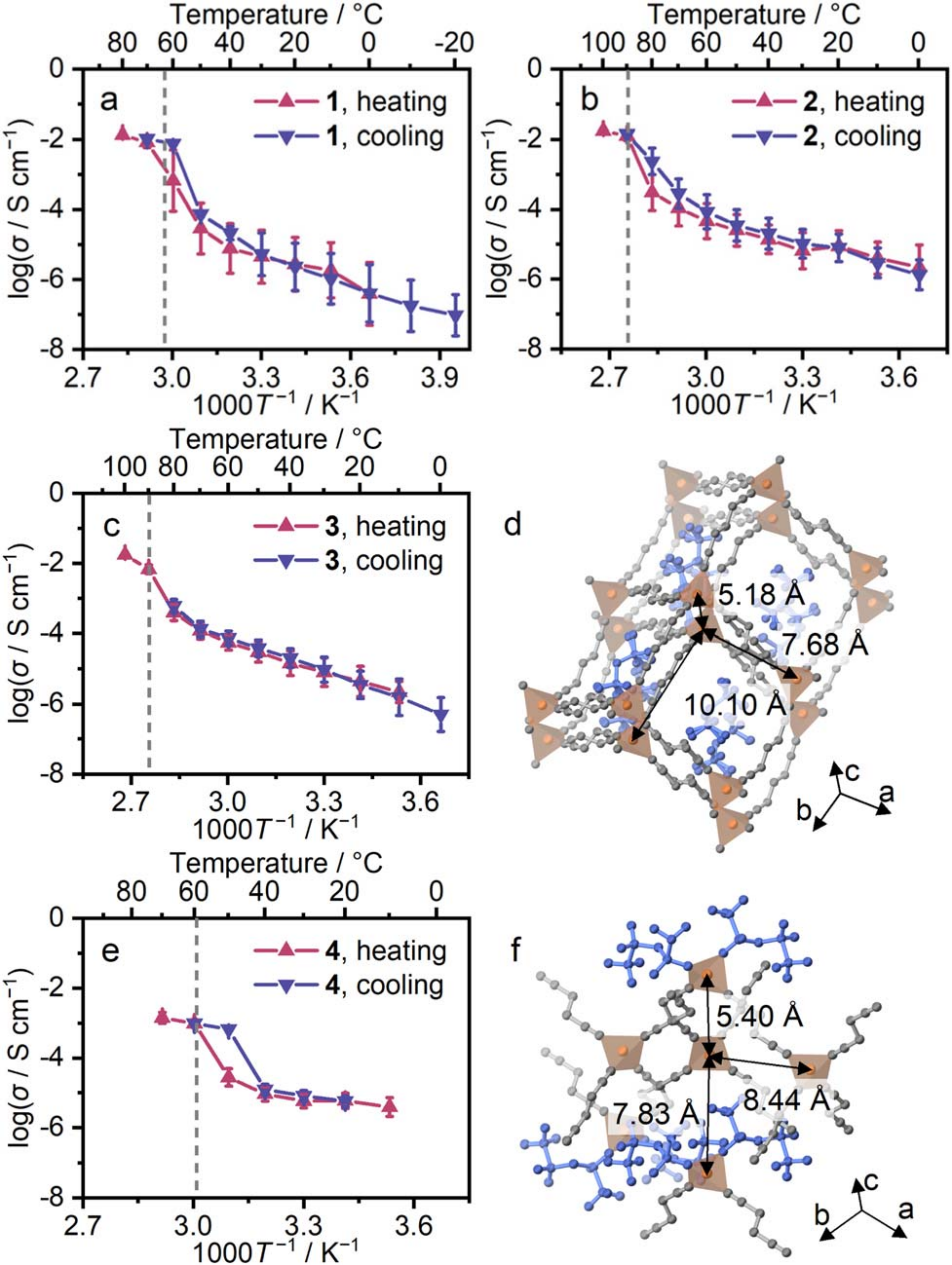
Li^+^ conductivity (*σ*) of 1–4. Arrhenius plots of Li^+^ conductivity for (a) 1, (b) 2, (c) 3, and (e) 4. Red and blue plots represent heating and cooling processes. The dashed lines indicate the *T*_m_. The crystal structures of (d) 3 and (f) 4. The distances between neighbouring Li^+^ are indicated.

Impedance measurements were conducted for 1–4 (Fig. S20 and S21). A high transference number (0.95) and a hopping conduction mechanism of Li^+^ were reported for 1, suggesting potential opportunities for Li^+^ conductivity in 2–4 as well.^10,37^ Temperature-step DSC was conducted for 4 with the same temperature program as the impedance measurements (Fig. S22), observing crystallisation at 50 °C. Ion conductivity was calculated from the semi-circles of the obtained Nyquist plot. Fig. 4 shows Arrhenius plots for the first and after-melting cycles. The conductivity (*σ*) of 1–4 at 30 °C in the after-melting cycle was 1.3, 1.5, 1.3, and 1.2 × 10^−5^ S cm^−1^, and the activation energies (*E*_a_) were 16, 13, 21, and 8 kJ mol^−1^ (Fig. S23). The comparable *σ* and *E*_a_ of 1–3 suggest the identical Li^+^ hopping mechanism in the conduction. The *E*_a_ of 4 is lower by 0.5 times, suggesting different Li^+^ conduction paths in 4. The crystal of 4 is in a lower symmetry with a higher number of neighbouring Li^+^ sites, and this leads to the formation of several possible Li^+^ ion paths, reducing the *E*_a_ in Li^+^ conduction (Fig. 4d and f). The ^7^Li *T*_1_ relaxation time in VT solid-state nuclear magnetic resonance (SS-NMR) corroborates the *E*_a_ of conductivity. In Fig. S24, the ^7^Li *T*_1_ relaxation time is plotted as a function of temperature. The *E*_a_ values of each ^7^Li *T*_1_ relaxation time followed the same trend as those observed from Li^+^ conductivity (Table S7). This suggests a Li^+^-conduction mechanism based on Li^+^ hopping, and the difference of *E*_a_ between 1–3 and 4 supports the identical Li^+^ hopping mechanism in 1–3 and multiple conduction paths in 4.

## Conclusions

Four framework crystals, Li(FSI)(SN)_2_, Li(FSI)(GN)_2_, Li(FSI)(SN)(GN), and Li(TFSI)(SN)_1.5_, were synthesised from Li^+^ and aliphatic dinitrile linkers by a solvent-free melt-cooling process. These are constructed through weak metal–ligand interactions, as explained by HSAB theory, leading to low *T*_m_ below 90 °C. DFT-based elasticity analyses revealed high and anisotropic deformability, attributed to the aliphatic softness and framework topology. XRD-based thermal expansion analyses observed a large linear CTE of +370 × 10^−6^ K^−1^ in one framework, as well as a large negative thermal expansion with a CTE of −110 × 10^−6^ K^−1^ in another one, attributed to structural anisotropy. AC impedance and solid-state NMR elucidated an ionic conductivity of 1.5 × 10^−5^ S cm^−1^ at room temperature based on the weak metal–linker interactions. This work provides clear evidence and guidelines for synthesising mechanically soft, thermally active molecular frameworks based on weak metal–linker interactions.

## Author contributions

T. N. and K. K. contributed equally to this work. T. N., K.·K., and S. H. conceptualised the project. K. K., T. N., T. K., N.·S., S. To., S. Ts, and N. M. contributed to data collection and formal analyses. T. N. and S. H. summarised the findings in the manuscript, and all the authors approved the final version.

## Conflicts of interest

There are no conflicts to declare.

## Supplementary Material

SC-017-D5SC06222E-s001

SC-017-D5SC06222E-s002

## Data Availability

CCDC 2463044 Li(FSI)(GN)_2_, 2463045 Li(FSI)(SN)(GN) and 2480466 Li(TFSI)(bpe)_2_ contain the supplementary crystallographic data for this paper.^38*a*–*c*^ The data supporting this article have been included in the supplementary information (SI). Supplementary information: the details of experiments, crystallographic data, computational analyses, thermogravimetric analysis, differential scanning calorimetry, variable-temperature powder X-ray diffraction and fitting, impedance and ion conductivity measurements, and solid-state nuclear magnetic resonance. See DOI: https://doi.org/10.1039/d5sc06222e.
