# Supplementary material for: Soft crystalline properties of 2D frameworks constructed from lithium ions and dinitriles
Source: Chem Sci. 2025 Dec 16;17(7):3669–74. doi: 10.1039/d5sc06222e (PMC12757750; doi:10.1039/d5sc06222e)
Supplement: SC-017-D5SC06222E-s001 [file SC-017-D5SC06222E-s001.pdf]

Supplementary Information (SI) for Chemical Science.
This journal is © The Royal Society of Chemistry 2025

Supporting Information for

Soft crystalline properties of 2D frameworks constructed from lithium ion and dinitriles

Taichi Nishiguchi,‡[a] Kotoha Kageyama,‡[b] Takuya Kurihara,[c] Nanae Shimanaka,[d] Shun Tokuda,[b,d] Shuto Tsuda,[a] Nattapol Ma[b] and Satoshi Horike*[a,d,e]

[a] Department of Chemistry, Graduate School of Science, Kyoto University, Kitashirakawa-Oiwakecho, Sakyo-ku, Kyoto 606-8502, Japan

[b] Department of Synthetic Chemistry and Biological Chemistry, Graduate School of Engineering, Kyoto University, Katsura, Nishikyo-ku, Kyoto 615-8510, Japan

[c] Division of Material Chemistry, Graduate School of Natural Science and Technology, Kanazawa University, Kakuma-Machi, Kanazawa, Ishikawa 920-1192, Japan

[d] Institute for Integrated Cell-Material Sciences, Institute for Advanced Study, Kyoto University, Yoshida-Honmachi, Sakyo-ku, Kyoto 606-8501, Japan

[e] Department of Materials Science and Engineering, School of Molecular Science and Engineering, Vidyasirimedhi Institute of Science and Technology, Rayong, 21210, Thailand

‡These authors contributed equally.

*e-mail: horike.satoshi.3r@kyoto-u.ac.jp

Table of contents

Methods 4

Materials 4

Syntheses and preparations 4

Material characterisations 5

Thermal characterisations 5

Ion conductivity measurements 5

Solid-state nuclear magnetic resonance (SS-NMR) 6

Computational analyses 6

Supplementary tables 7

Table S1. Crystallographic data of **2**–**4** and Li(TFSI)$(bpe)_2$ 7

Table S2. Extracted cell parameters and refinement results for the PXRD patterns of **1**+**2** mixture. 7

Table S3. Extracted cell parameters and refinement results for the PXRD patterns of $Li(FSI)_{0.5}(TFSI)_{0.5}(GN)_2$ 7

Table S4. Extracted cell parameters and refinement results for the VT-PXRD patterns of **1**. 8

Table S5. Extracted cell parameters and refinement results for the VT-PXRD patterns of **2**. 8

Table S6. Extracted cell parameters and refinement results for the VT-PXRD patterns of **3**. 8

Table S7. Activation energy of ^1H and ^7Li T_1 relaxation in SS-NMR of **1**–**4**. 8

Supplementary figures 9

Figure S1. Crystal structures of (viewed along) (a) 1 (*a*-axis), (b) 1 (*b*-axis), (c) 2 (*a*-axis), (d) 2 (*b*-axis), (e) 3 (*c*-axis), (f) 3 (*b*-axis), (g) 4 (*a*-axis), (h) 4 (*b*-axis), and (i) 4 (*c*-axis). 9

Figure S2. TG-DTA of (a) **1**, (b) **2**, (c) **3**, and (d) **4**. 10

Figure S3. VT-PXRD patterns of (a) **1**, (b) **1** at −100 °C (enlarged), (c) **2**, (d) **2** at −30 °C (enlarged), (e) **3**, and (f) **4**. 11

Figure S4. Crystal structure of Li(TFSI)$(bpe)_2$ viewed along (a) *c*-axis, (b) *a*-axis, and (c) *b*-axis. 12

Figure S5. (a) TG-DTA and (b) DSC profile of Li(TFSI)$(bpe)_2$. 12

Figure S6. DSC profiles of **4** with different temperature-ramping rates. 12

Figure S7. PXRD pattern of **1**+**2** physical mixture. A pattern of **3** is shown for comparison. 13

Figure S8. PXRD pattern and Pawley fit of **1**+**2** mixture. 13

Figure S9. VT-PXRD patterns of (a) $\mathbf{1_{0.25}4_{0.75}}$, (b) $\mathbf{1_{0.5}2_{0.5}}$, (c) $\mathbf{1_{0.75}4_{0.25}}$. 14

Figure S10. DSC profiles of $\mathbf{1}_x\mathbf{4}_{1-x}$. 14

Figure S11. DSC profile of $Li(FSI)_{0.5}(TFSI)_{0.5}(GN)_2$. 15

Figure S12. PXRD pattern of $Li(FSI)_{0.5}(TFSI)_{0.5}(GN)_2$. The pattern of **2** is shown for comparison. 15

Figure S13. PXRD patterns and Pawley fit of $Li(FSI)_{0.5}(TFSI)_{0.5}(GN)_2$. 16

Figure S14. DSC curve of Li(TFSI)$(GN)_2$. 16

Figure S15. VT-PXRD patterns and Pawley fit of **1** at (a) −50, (b) 30, (c) 40, (d) 50, and (e) 55 °C. 17

Figure S16. VT-PXRD patterns and Pawley fit of **2** at (a) 0, (b) 30, (c) 60, (d) 70, and (e) 80 °C. 18

Figure S17. VT-PXRD patterns and Pawley fit of **3** at (a) −150, (b) 0, (c) 30, (d) 60, (e) 70, and (f) 80 °C. 19
Figure S18. DFT-optimised geometry of (a) **1**, (b) **2**, and (c) **3** employed in the computational mechanical analyses. .20
Figure S19. Elastic tensors of (a) **1**, (b) **2**, and (c) **3**, obtained in the computational mechanical analyses in the unit of GPa. 20
Figure S20. Nyquist plots of (a–c) heating process of **1**, (d, e) cooling process of **1**, (f–i) heating process of **2**, (j–l) cooling process of **2**. 21
Figure S21. Nyquist plots of (a–d) heating process of **3**, (e–g) cooling process of **3**, (h–j) heating process of **4**, (k–m) cooling process of **4**. 22
Figure S22. Temperature-step and isothermal DSC profile of **4**. The blue line represents the heat flow, and the red line represents the temperature program. Asterisk (*) indicates the crystallisation exotherm........23
Figure S23. Arrhenius plots of conductivity with temperature-weighted conductivity of (a) **1**, (b) **2**, (c) **3**, and (d) **4** in the heating processes for determination of the activation energies. The dashed lines indicate the fitting results. 23
Figure S24. ^{7}Li relaxation time of **1** (red), **2** (yellow), **3** (pink), and **4** (blue). The solid lines indicate the fitting curves. 24

References 25

Methods

Materials

Lithium bis(fluorosulfonyl)imide (> 98.0%, Li(FSI)), lithium bis(trifluoromethanesulfonyl)imide (> 98.0%, Li(TFSI)), glutaronitrile (> 96.0%, GN), succinonitrile (> 99.0%, SN), and 1,2-bis(4-pyridyl)ethane (> 98%, bpe) were purchased from Tokyo Chemical Industry Co., Ltd. All chemicals were in the reagent grade and used without any further purification.

Syntheses and preparations

All synthetic processes were done under an argon atmosphere to avoid moisture.

Synthesis of Li(FSI)(SN)$_2$ (1): 1 was synthesised according to a reported method.[1] Li(FSI) (0.935 g, 5.0 mmol) and SN (0.801 g, 10.0 mmol) were added into a 10 mL vial and heated at 72 °C for 2 hours with stirring. The mixture was cooled to room temperature to obtain **1** as a colourless solid.

Synthesis of Li(FSI)(GN)$_2$ (2): Li(FSI) (0.935 g, 5.0 mmol) and GN (0.941 g, 10.0 mmol) were added into a 10 mL vial and heated at 95 °C for 2 hours with stirring. The mixture was cooled to room temperature to obtain **2** as a colourless solid. A single crystal suitable for SC-XRD analysis was picked from the obtained solid.

Synthesis of Li(FSI)(SN)(GN) (3): Li(FSI) (0.935 g, 5.0 mmol), SN (0.401 g, 5.0 mmol), and GN (0.471 g, 5.0 mmol) were added into a 10 mL vial and heated at 115 °C for 2 hours with stirring. The mixture was cooled to room temperature to obtain **3** as a colourless solid. A single crystal suitable for SC-XRD analysis was picked from the obtained solid.

Synthesis of Li(TFSI)(SN)$_{1.5}$ (4): Li(TFSI) (1.435 g, 5.0 mmol) and SN (0.601 g, 7.5 mmol) were added into a 10 mL vial and heated at 72 °C for 2 hours with stirring. The mixture was cooled to room temperature to obtain **4** as a colourless solid. A single crystal suitable for SC-XRD analysis was picked from the obtained solid.

Synthesis of Li(TFSI)(bpe)$_2$: Li(TFSI) (1.435 g, 5.0 mmol) and bpe (1.842 g, 10.0 mmol) were added into a 10 mL vial and heated at 250 °C for 10 minutes with stirring. The mixture was cooled to room temperature to obtain a colourless solid.

Preparation of $\mathbf{1}_x\mathbf{4}_{1-x}$ (x = 0.25, 0.5, 0.75): Li(FSI) (1.0/2.0/3.0 mmol), Li(TFSI) (3.0/2.0/1.0 mmol), and SN (6.5/7.0/7.5 mmol) were added into a 10 mL vial and heated at 100 °C for 2 hours with stirring. The mixture was cooled to room temperature to obtain a colourless solid.

Preparation of Li(FSI)$_{0.5}$(TFSI)$_{0.5}$(GN)$_2$: Li(FSI) (0.463 g, 2.5 mmol), Li(TFSI) (0.718 g, 2.5 mmol), and GN (0.941 g, 10.0 mmol) were added into a 10 mL vial and heated at 100 °C for 2 hours with stirring. The mixture was cooled to room temperature to obtain a colourless solid.

Preparation of Li(TFSI)(SN)(GN): Li(TFSI) (1.435 g, 5.0 mmol), SN (0.401 g, 5.0 mmol), and GN (0.471 g, 5.0 mmol) were added into a 10 mL vial and heated at 72 °C for 2 hours with stirring. The mixture was cooled to room temperature to obtain a colourless liquid.

Preparation of Li(TFSI)(GN)$_2$: Li(TFSI) (1.435 g, 5.0 mmol) and GN (0.941 g, 10.0 mmol) were added into a 10 mL vial and heated at 72 °C for 2 hours with stirring. The mixture was cooled to room temperature to obtain a colourless liquid.

Material characterisations

The single-crystal X-ray diffraction (SC-XRD) measurements were conducted with a Rigaku XtaLab P200 diffractometer and a Dectris Pilatus 200K system at 0 °C (for **2** and **3**) or at −175 °C (for **4**). The system is equipped with a MicroMax007 HF/VariMax rotating-anode X-ray generator with confocal monochromatised Mo-Kα radiation. The SC-XRD measurements of Li(TFSI)$(bpe)_2$ were performed at 100 K with Cu-Kα radiation. The collected data were solved by a direct method (ShelXT 2018/2) and refined by full-matrix least-squares refinement using SHELXL 2019/3.[2,3] Variable-temperature (VT) powder X-ray diffracton (PXRD) patterns of **1**–**4**, **1**+**2** mixture, $Li(FSI)_{0.5}(TFSI)_{0.5}(GN)_2$ were obtained at BL02B2 at the Super Photon ring-8 GeV (SPring-8, Hyogo, Japan) with Si detectors. Each sample was sealed in a Lindemann glass capillary with a diameter of 0.4 mm under an Ar atmosphere. The temperature was ramped at the rate of 30 °C min^{-1}. The exposure time for data collection was set to 30 seconds at each temperature. The temperature ranges were −100 to 60 °C (for **1**) or −100 to 100 °C (for **2** and **3**), considering each T_m. Pawley fitting method was applied to the obtained VT-PXRD patterns with Rigaku PDXL software to analyse thermal expansivity.[4] For $\mathbf{1}_x\mathbf{4}_{1-x}$, VT-PXRD data were collected at BL04B2, SPring-8 with CdTe detectors. Each sample was sealed in a borosilicate glass capillary with a diameter of 0.7 mm under an Ar atmosphere. The temperature was ramped at the rate of 30 °C min^{-1}. The exposure time for data collection was set to 30 seconds at each temperature.

Thermal characterisations

Thermogravimetric analysis (TGA) and differential thermal analysis (DTA) results were collected using a Rigaku Thermo plus TG 8121 apparatus with a heating rate of 10 °C min^{-1} under an Ar atmosphere inside an Ar-filled glove box. Samples were mounted in Al TGA pans. Decomposition temperature was determined as the temperature at 2% of weight loss. Differential scanning calorimetry (DSC) data were collected by a Hitachi DSC 7020. Samples were sealed in Al pans under an Ar atmosphere. The measurements were conducted between the following temperatures with a heating and cooling rate of 10 °C min^{-1}. Temperature-step measurement of **4** was performed with the ramping rate of 10 K min^{-1} with the steps of 70, 60, 50, and 40 °C for 2 hours, respectively.

Ion conductivity measurements

Ion conductivity measurements were performed by the alternating-current (AC) impedance spectroscopy technique. The electrochemical impedance spectroscopies were conducted using a BioLogic VSP-300 over the frequency range from 1 MHz to 100 mHz with an input voltage amplitude of 30 mV. Samples were filled in a cell of EC Frontier between two Pt sheets as electrodes. All samples were measured under an Ar atmosphere. The conductivity depending on temperature was obtained from −20 to 80 °C for **1**, from 0 to 100 °C for **2** and **3**, and from 10 to 70 °C for **4** to ensure the melting of the samples. The temperature was swept by 10 °C of step, and in each step, 2 hours of waiting was set for thermal equilibrium before measurements.

Collected data were converted to Arrhenius plots by fitting them to the equivalent circuit using ZView software. The ion conductivity (σ) was calculated based on the equation $\sigma = l/SR$, where R is the bulk resistance, S is the area of the surface of the hole of the EC cell, and l is the thickness of the electrolyte material. The bulk resistance ($1/\sigma$) was calculated from the equivalent circuit fitting, which is R_1 in the circuit of R_0–(R_1–Q_1)–(R_2–Q_2) (R: resistance, Q: constant phase element). The conductivity was determined as the average over cycles of impedance measurements

with error bars of a confidence of 95% for heating and cooling processes each.

The activation energy (E_a) of conductivity was obtained from each Arrhenius plot of heating processes. Each conduction data point was plotted into a T^{-1} vs. $\ln(\sigma T)$ graph. E_a was obtained as the slope and Boltzmann constant (k_B) after fitting the line by least-square method with OriginPro software for the temperature range of 10–40 °C after melting.

$$\sigma = \frac{A}{T} \mathrm{e}^{-\frac{E_a}{k_B T}}$$

Solid-state nuclear magnetic resonance (SS-NMR)

^{1}H and ^{7}Li SS-NMR experiments were performed on a JEOL JNM-ECZ600R spectrometer at 14.1 T (^{1}H 600.17 MHz) with a JEOL 3.2 mm double resonance MAS probe. VT ^{1}H and ^{7}Li T_1 relaxation time measurements were conducted under no sample spinning condition.

The temperature dependences of ^{1}H T_1 of **2** and ^{7}Li T_1 of **2**, **3**, and **4** were fitted using BPP equations,[5]

$$\frac{1}{T_1^{1\mathrm{H}}} = D_{\mathrm{H-H}} \left\{ \frac{2\tau_c}{1+\omega_{0\mathrm{H}}^2\tau_c^2} + \frac{8\tau_c}{1+(2\omega_{0\mathrm{H}})^2\tau_c^2} \right\}$$

and

$$\frac{1}{T_1^{7\mathrm{Li}}} = D_{\mathrm{Li-H}} \left\{ \frac{2\tau_c}{1+(\omega_{0\mathrm{H}}-\omega_{0\mathrm{Li}})^2\tau_c^2} + \frac{6\tau_c}{1+\omega_{0\mathrm{Li}}^2\tau_c^2} + \frac{12\tau_c}{1+(\omega_{0\mathrm{H}}+\omega_{0\mathrm{Li}})^2\tau_c^2} \right\},$$

with the assumption that the dominant relaxation mechanisms are ^{1}H–^{1}H homonuclear and ^{7}Li–^{1}H heteronuclear dipolar interactions, respectively. $D_{\mathrm{X-H}}$ (X = H, Li) is a constant depending on the dipolar coupling between X and ^{1}H nuclei and molecular dynamics, $\omega_{0\mathrm{X}}$ is the Larmor frequency of X, and τ_c is the correlation time of a motion expressed as the Arrhenius law $\tau_c = \tau_0 \exp(E_a / RT)$, where τ_0 is the pre-exponential factor, E_a is the activation energy, R is the universal gas constant, and T is temperature. The ^{7}Li of **1** and ^{1}H T_1 relaxation behaviours of **1**, **3**, and **4** were fitted using an Arrhenius-type equation because the local minimum of T_1 was not observed in these temperature ranges. The ^{1}H and ^{7}Li T_1 fitting analyses of **2** were performed above T_{ss} (−12 °C). Both ^{1}H and ^{7}Li T_1 values at −20 °C do not match the fitting functions, indicating the change of the ligand mobility below T_{ss}.

Computational analyses

For crystalline systems, we used Quantum ESPRESSO package.[6,7] Geometry and the cell parameters were optimised based on the structures obtained from SC-XRD analyses. We used PBE functional and PAW-type plane-wave basis sets with pseudo-potentials. The calculation was performed for the 1 × 1 × 2 k point grid. The calculation was performed with PWscf program with ElaStic package, and the obtained data was analysed by ELATE program.[8,9] For all the computational studies, we used the machine time of supercomputer systems provided by the Institute for Chemical Research (ICR), Kyoto University, Japan.

Supplementary tables

Table S1. Crystallographic data of **2**–**4** and Li(TFSI)(bpe)$_2$

Compound	Li(FSI)(GN)$_2$ (**2**)	Li(FSI)(SN)(GN) (**3**)	Li(TFSI)(SN)$_{1.5}$ (**4**)	Li(TFSI)(bpe)$_2$
CCDC deposit number	2463044	2463045	*	2480466
Formula	$Li_1C_{10}H_{12}N_5O_4F_2S_2$	$Li_1C_9H_{10}N_5O_4F_2S_2$	$Li_1C_8H_6N_4O_4F_6S_2$	$Li_1C_{26}H_{24}N_5O_4F_6S_2$
Formula weight	375.30	361.27	407.22	655.56
Colour	Colourless	Colourless	Colourless	Colourless
Temperature / K	273	273	98	100
Wavelength / Å	0.71073 (Mo Kα)	0.71073 (Mo Kα)	0.71073 (Mo Kα)	1.54184 (Cu Kα)
Theta range / °	2.3430 – 19.9950	2.8170 – 27.5910	2.5180 – 30.4260	2.9280 – 76.7630
Crystal system	Orthorhombic	Orthorhombic	Triclinic	Tetragonal
Space group	$P2_12_12$	*Pbcm*	P–1	P–4
a / Å	12.8522(6)	5.1756(4)	11.9957(4)	21.3025(1)
b / Å	12.8465(6)	15.2002(13)	12.2939(4)	21.3025(1)
c / Å	5.2547(3)	20.2029(15)	13.1343(4)	6.4513(1)
α / °	90	90	101.365(3)	90
β / °	90	90	106.245(3)	90
γ / °	90	90	114.359(3)	90
V / Å^3	867.59(8)	1589.4(2)	1582.3	2927.58(5)
Z	2	4	2	4
ρ / g cm^{-3}	1.437	1.510	1.710	1.487
μ / mm^{-1}	0.350	0.379	0.425	2.373
Number of collected/ independent reflection	14670/2719	11720/2473	62418/9655	17018/5614
R_1, wR_2 [$I > 2\sigma(I)$]	0.0368, 0.0783	0.0630, 0.1841	0.0359, 0.0849	0.0261, 0.0703
R_1, wR_2 (all data)	0.0962, 0.0995	0.0892, 0.2039	0.0515, 0.0909	0.0278, 0.0716
Goodness of fit	1.016	1.072	1.029	1.044

*A crystallographic data set of the same compound was deposited with the number 2361840 before our submission.[10]

Table S2. Extracted cell parameters and refinement results for the PXRD patterns of **1**+**2** mixture.

Temperature / °C	a / Å	b / Å	c / Å	R_{wp}	R_p	R_e	S
30	5.2380(2)	15.3480(6)	20.2381(9)	0.0591	0.0352	0.0372	1.5889

Table S3. Extracted cell parameters and refinement results for the PXRD patterns of Li(FSI)$_{0.5}$(TFSI)$_{0.5}$(GN)$_2$.

Temperature / °C	a / Å	b / Å	c / Å	R_{wp}	R_p	R_e	S
30	12.9159(3)	12.9159(3)	5.30875(13)	0.0441	0.0314	0.0311	1.4195

Table S4. Extracted cell parameters and refinement results for the VT-PXRD patterns of **1**.

Temperature / °C	a / Å	b / Å	c / Å	R_{wp}	R_p	R_e	S
−50	12.1072(3)	12.1072(3)	4.99692(15)	0.0602	0.0339	0.0209	2.8727
30	12.03674(16)	12.03674(16)	5.14583(7)	0.0780	0.0456	0.0350	2.2309
40	12.0284(4)	12.0284(4)	5.16484(18)	0.0744	0.0435	0.0350	2.1235
50	12.02265(8)	12.02265(8)	5.18327(4)	0.0961	0.0519	0.0350	2.7450
55	12.02002(15)	12.02002(15)	5.19155(7)	0.0707	0.0422	0.0339	2.0848

Table S5. Extracted cell parameters and refinement results for the VT-PXRD patterns of **2**.

Temperature / °C	a / Å	b / Å	c / Å	R_{wp}	R_p	R_e	S
0	12.8813(3)	12.8722(3)	5.27219(12)	0.0506	0.0275	0.0203	2.7564
30	12.9040(5)	12.9010(5)	5.30225(7)	0.0575	0.0356	0.0291	1.9774
60	12.93378(12)	12.92306(17)	5.33267(6)	0.0439	0.0302	0.0287	1.5292
70	12.9402(17)	12.9396(17)	5.34425(8)	0.0462	0.0298	0.0287	1.6097
80	12.9523(7)	12.9503(7)	5.35480(6)	0.0374	0.0268	0.0295	1.2676

Table S6. Extracted cell parameters and refinement results for the VT-PXRD patterns of **3**.

Temperature / °C	a / Å	b / Å	c / Å	R_{wp}	R_p	R_e	S
−100	5.09414(16)	15.0496(5)	20.3248(5)	0.0904	0.0549	0.0296	3.0599
0	5.18899(7)	15.22916(18)	20.2315(2)	0.0869	0.0479	0.0325	2.6735
30	5.2305(3)	15.3154(8)	20.1260(9)	0.0688	0.0419	0.0293	2.3454
60	5.30255(17)	15.4738(5)	19.9621(5)	0.1144	0.0607	0.0294	3.8880
70	5.32943(8)	15.5097(2)	19.92436(12)	0.0573	0.0382	0.0294	1.9480
80	5.34355(7)	15.5251(2)	19.90564(11)	0.0536	0.0360	0.0291	1.8437

Table S7. Activation energy of ^{1}H and ^{7}Li T_1 relaxation in SS-NMR of **1**–**4**

Sample	T range of T_1 analysis / °C	^{1}H motional rate (30 °C) / s^{-1}	^{1}H E_a / kJ mol^{-1}	^{7}Li motional rate (30 °C) / s^{-1}	^{7}Li E_a / kJ/mol
1	0 to 50	$> 10^{10}$	3.6	$> 10^{10}$	13.1
2	−10 to 80	5.8×10^{9}	16.5	4.2×10^{9}	22.6
3	−20 to 30	$> 10^{10}$	3.8	4.3×10^{9}	18.6
4	−70 to 50	$> 10^{10}$	2.7	2.5×10^{9}	7.7

Supplementary figures

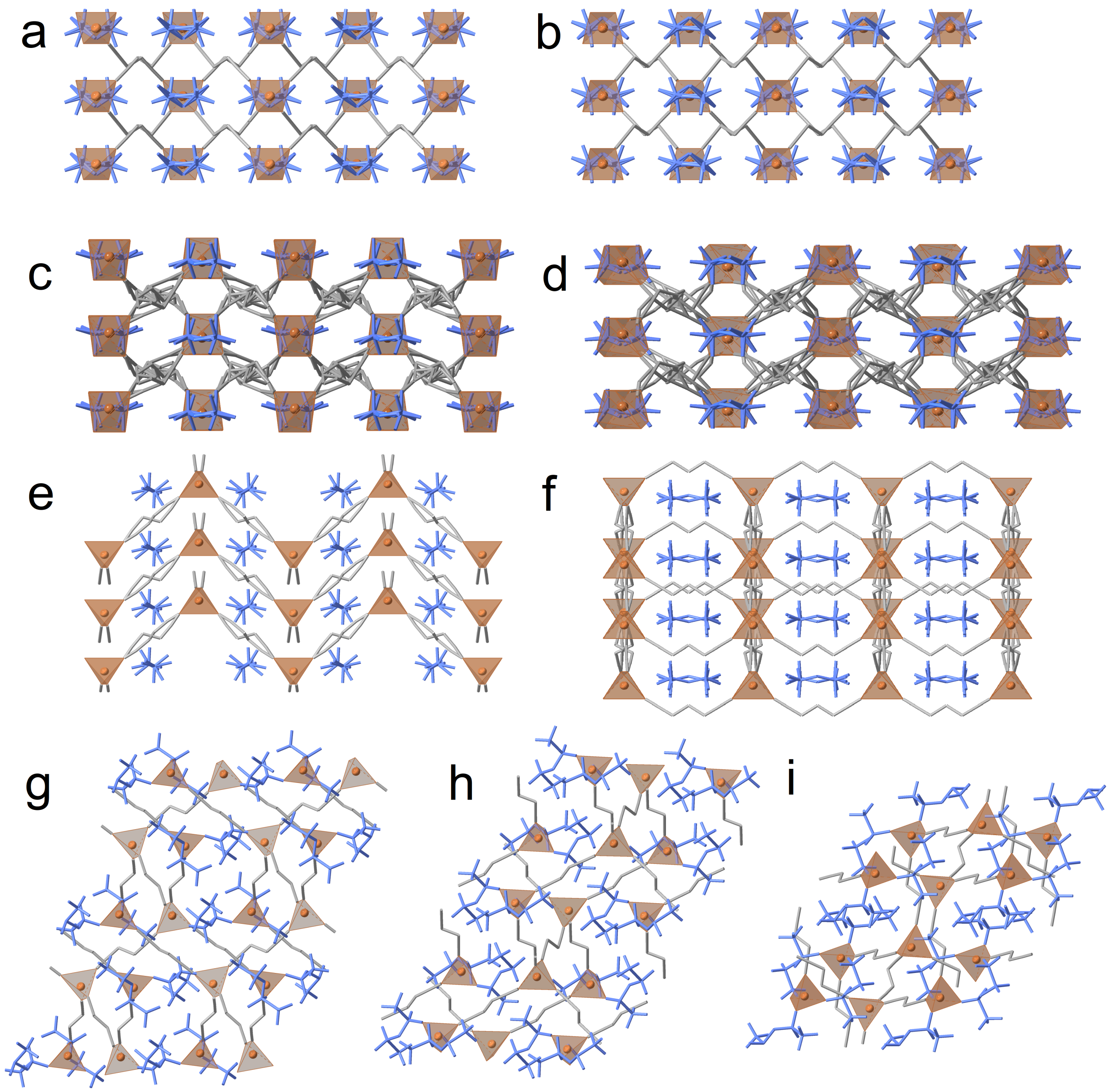

Figure S1. Crystal structures of (viewed along) (a) 1 (*a*-axis), (b) 1 (*b*-axis), (c) 2 (*a*-axis), (d) 2 (*b*-axis), (e) 3 (*c*-axis), (f) 3 (*b*-axis), (g) 4 (*a*-axis), (h) 4 (*b*-axis), and (i) 4 (*c*-axis).

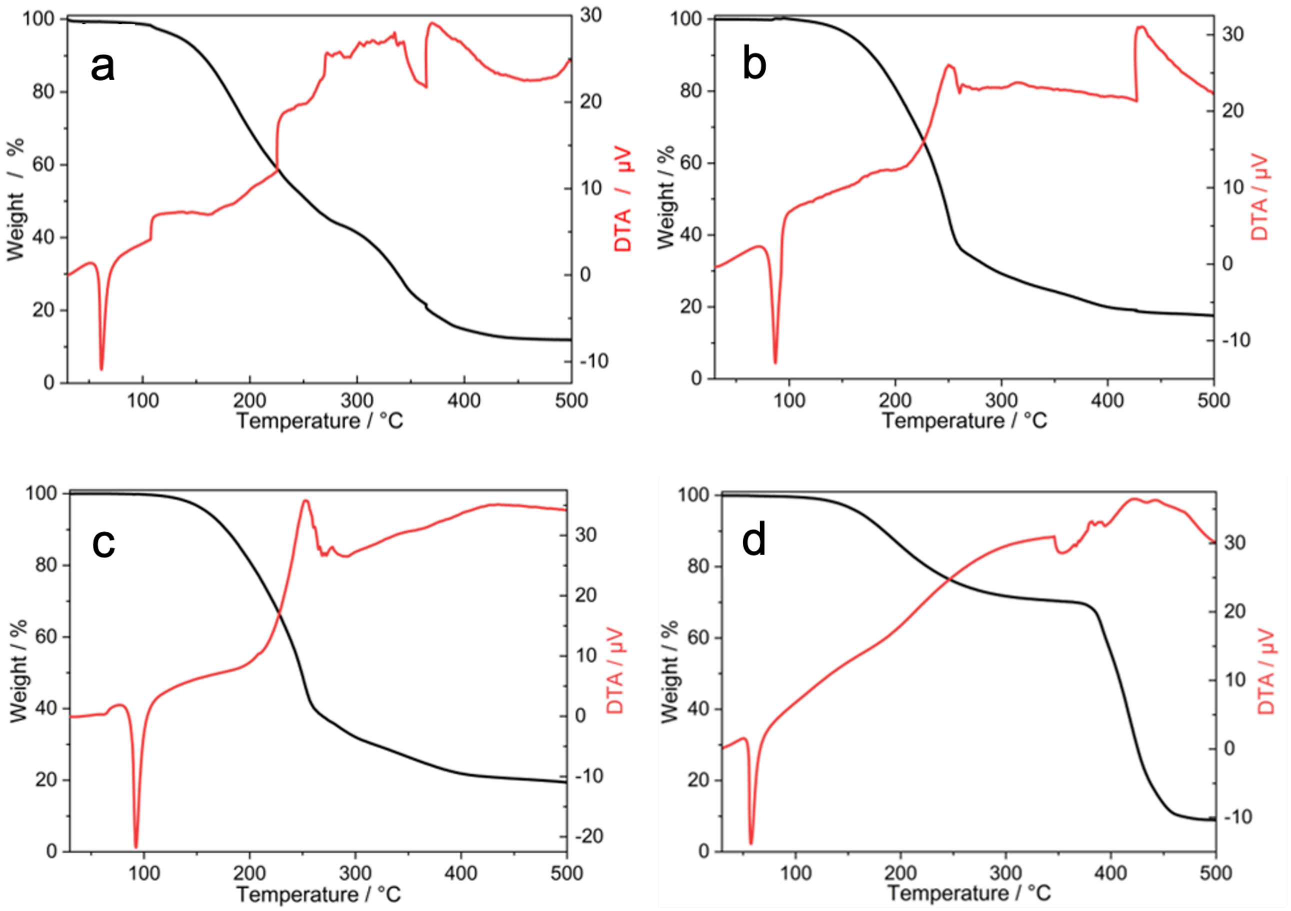

Figure S2. TG-DTA of (a) **1**, (b) **2**, (c) **3**, and (d) **4**.

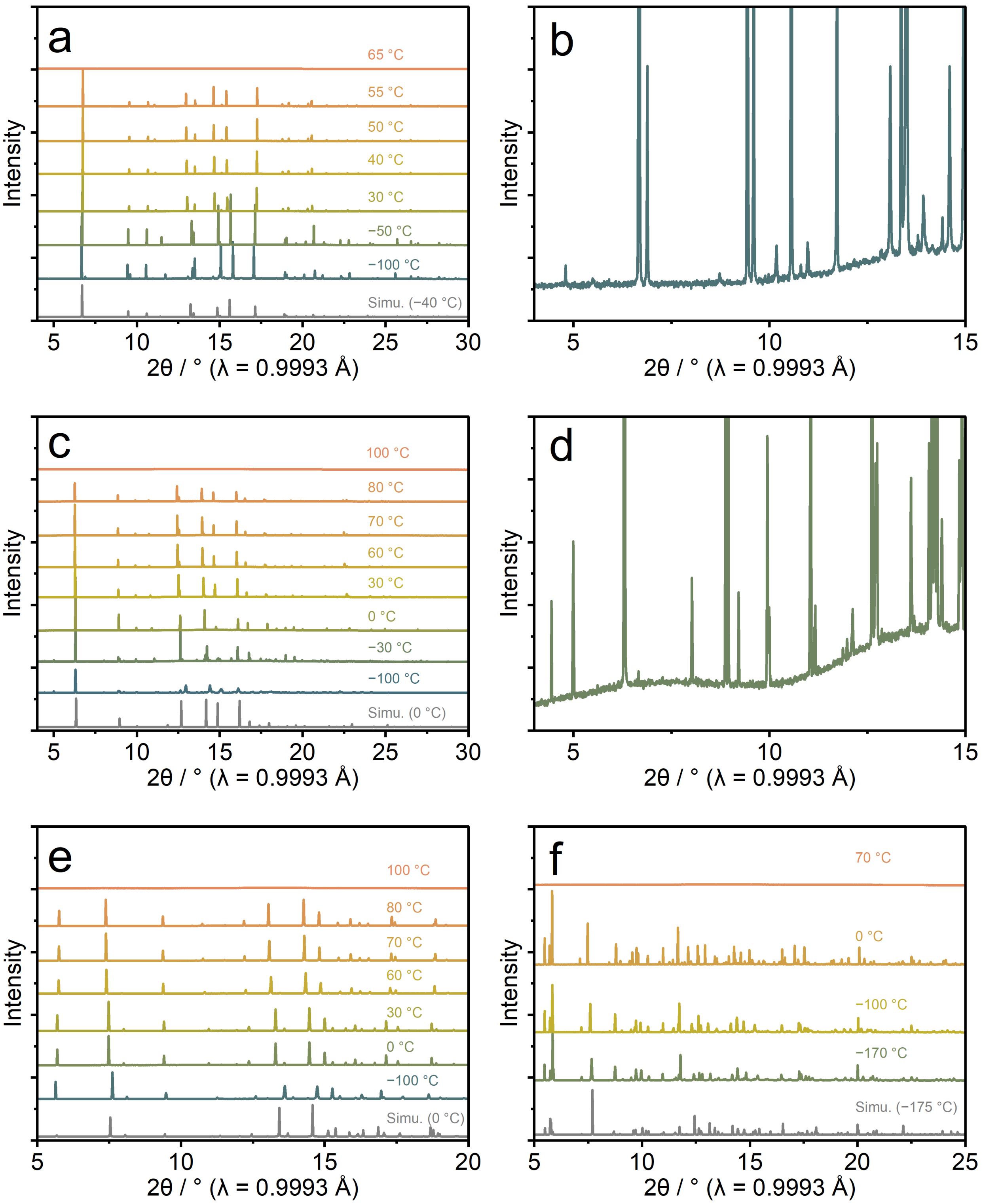

Figure S3. VT-PXRD patterns of (a) **1**, (b) **1** at −100 °C (enlarged), (c) **2**, (d) **2** at −30 °C (enlarged), (e) **3**, and (f) **4**.

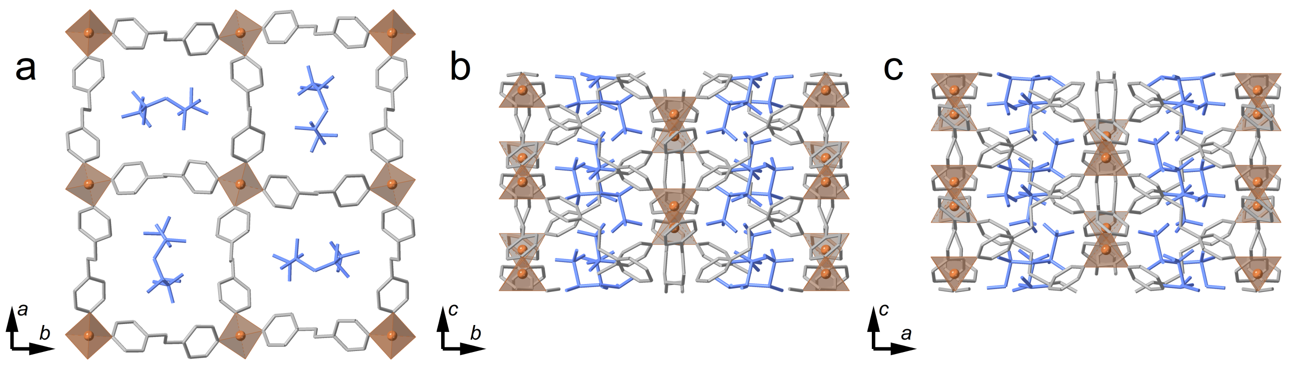

Figure S4. Crystal structure of Li(TFSI)(bpe)$_2$ viewed along (a) *c*-axis, (b) *a*-axis, and (c) *b*-axis.

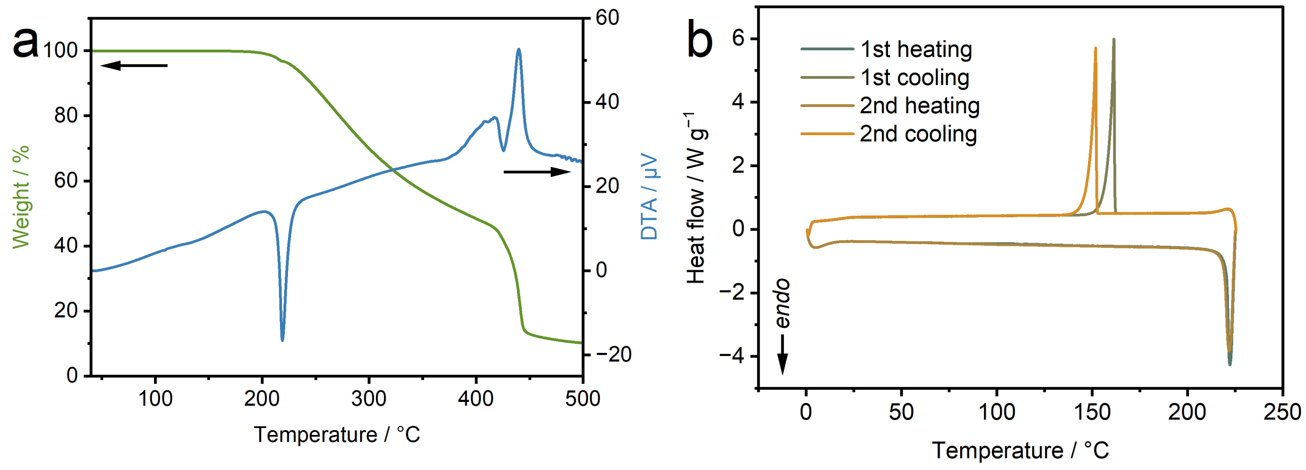

Figure S5. (a) TG-DTA and (b) DSC profiles of Li(TFSI)(bpe)$_2$.

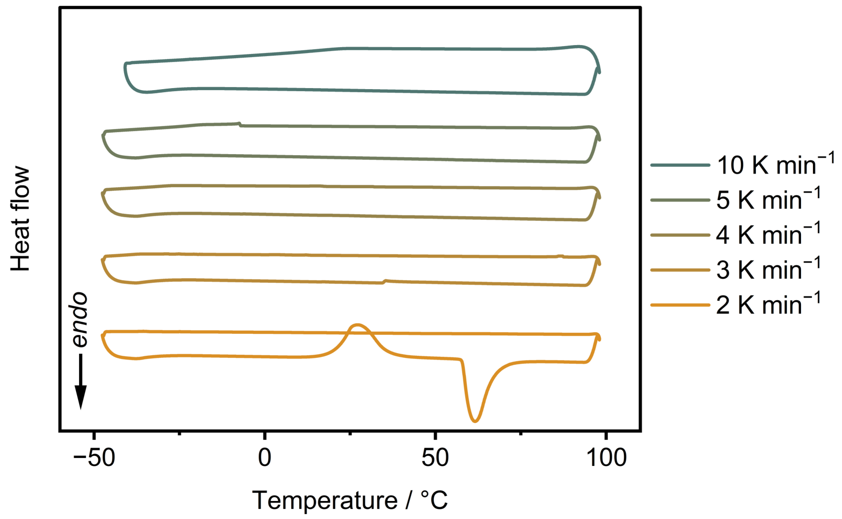

Figure S6. DSC profiles of **4** with different temperature-ramping rates.

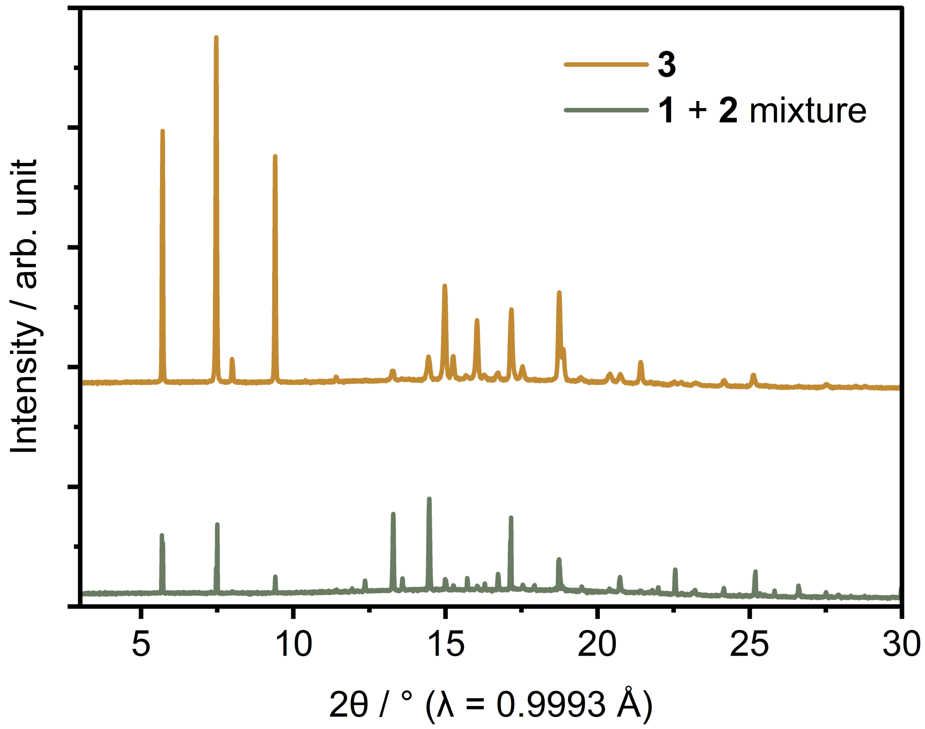

Figure S7. PXRD pattern of **1**+**2** physical mixture. A pattern of **3** is shown for comparison.

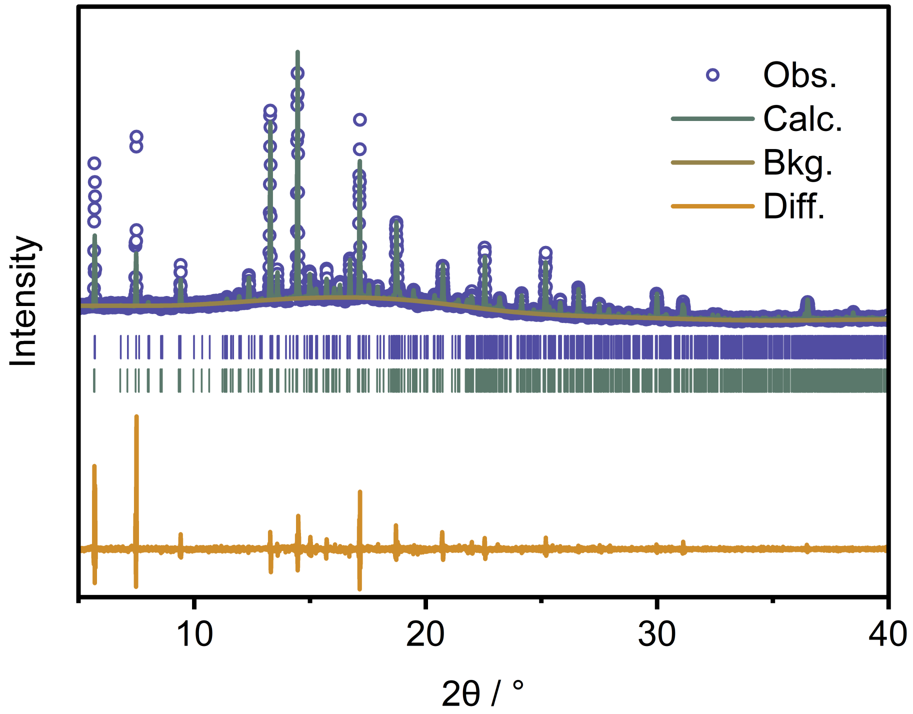

Figure S8. PXRD pattern and Pawley fit of **1**+**2** mixture.

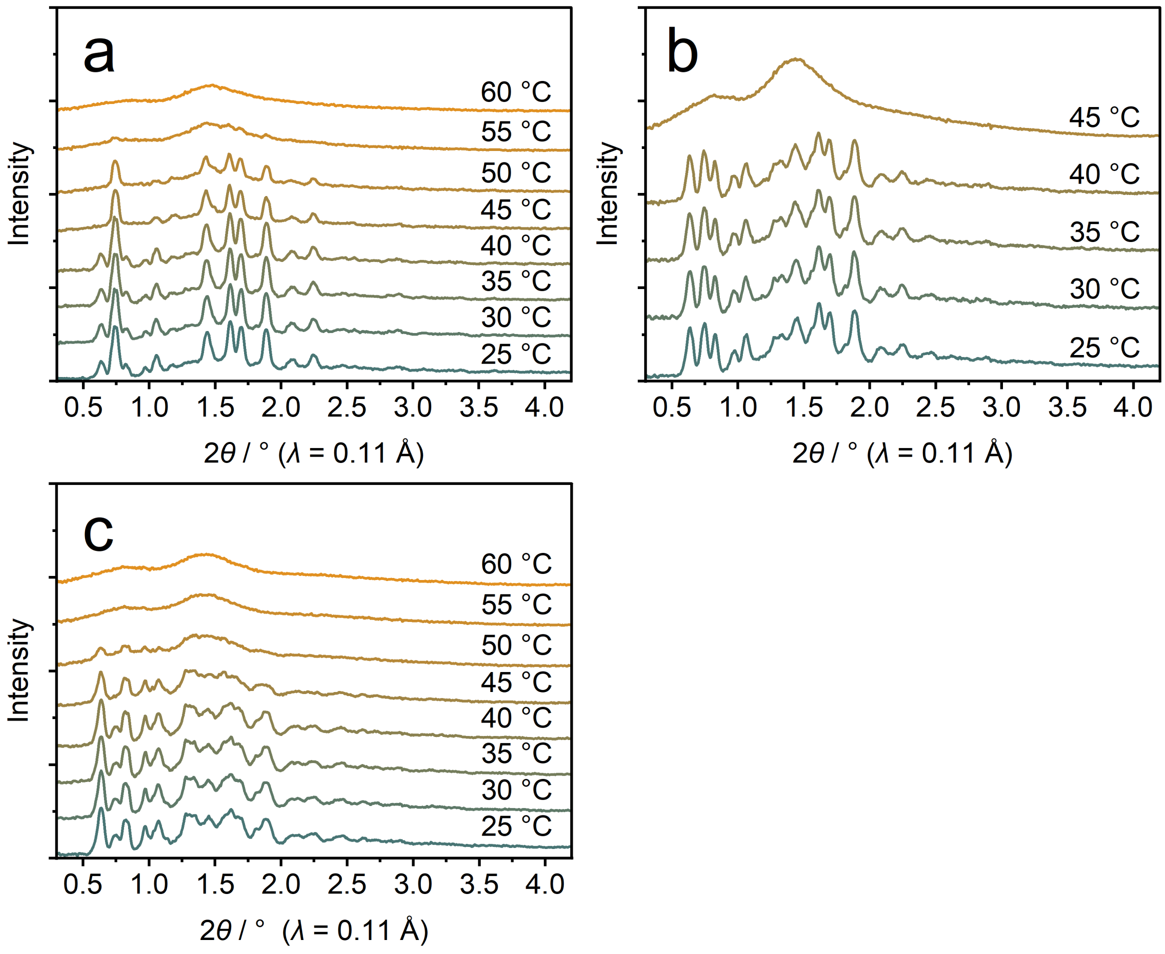

Figure S9. VT-PXRD patterns of (a) $\mathbf{1}_{0.25}\mathbf{4}_{0.75}$, (b) $\mathbf{1}_{0.5}\mathbf{2}_{0.5}$, (c) $\mathbf{1}_{0.75}\mathbf{4}_{0.25}$.

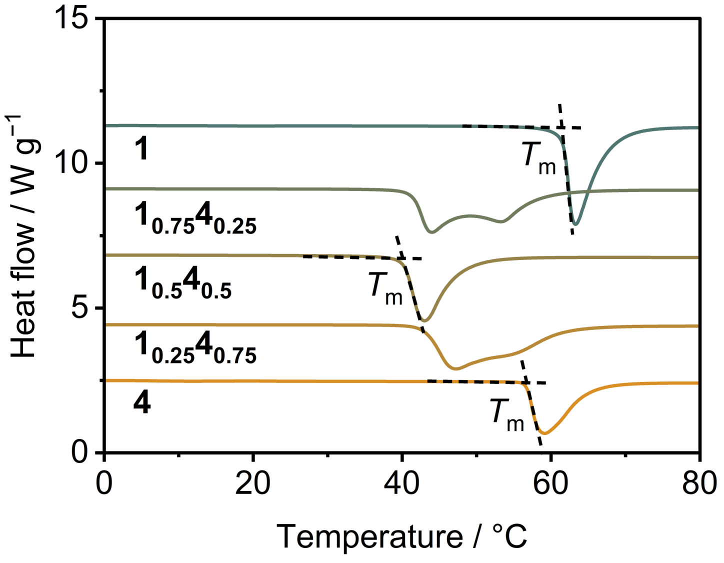

Figure S10. DSC profiles of $\mathbf{1}_{x}\mathbf{4}_{1-x}$.

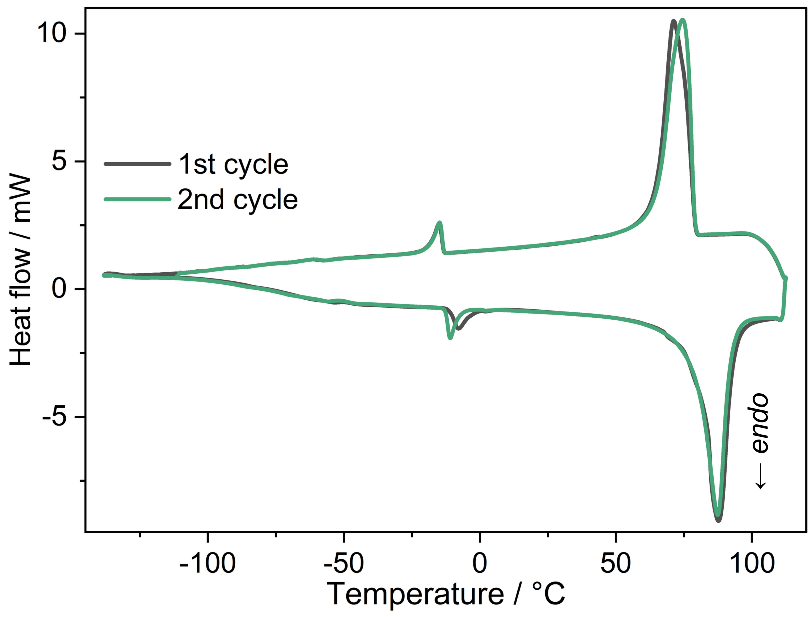

Figure S11. DSC profile of $Li(FSI)_{0.5}(TFSI)_{0.5}(GN)_2$.

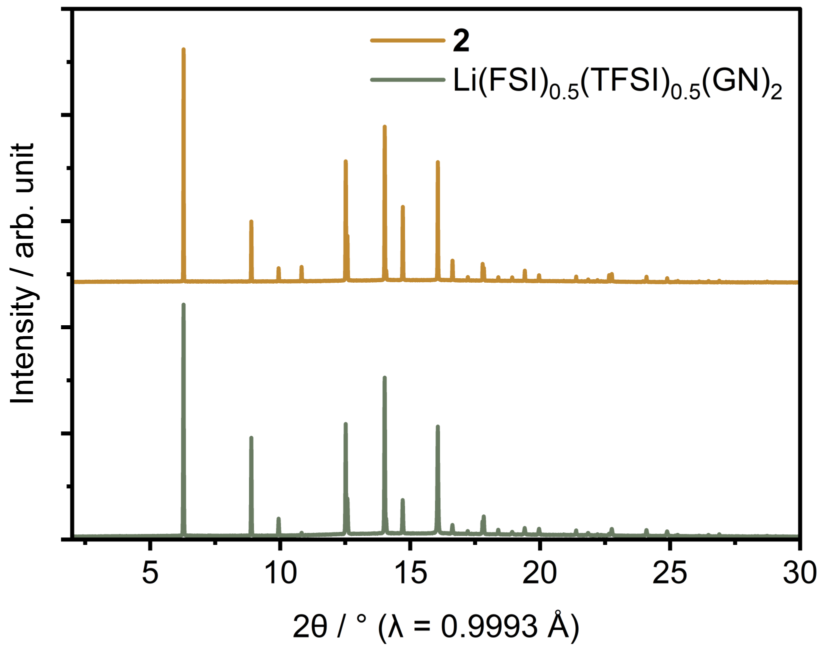

Figure S12. PXRD pattern of $Li(FSI)_{0.5}(TFSI)_{0.5}(GN)_2$. The pattern of **2** is shown for comparison.

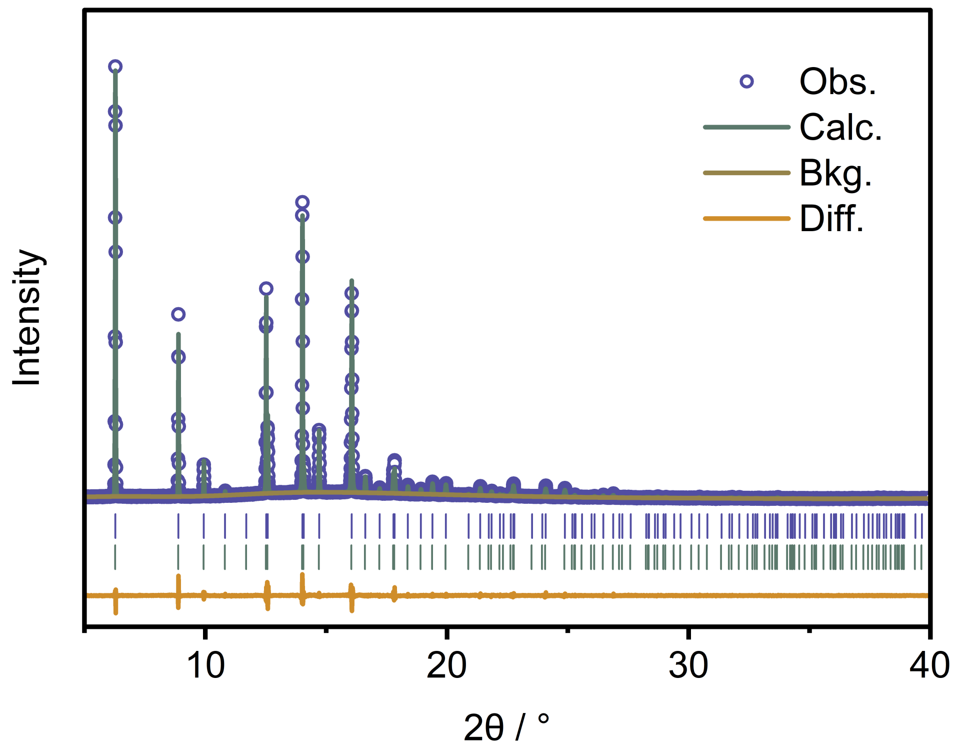

Figure S13. PXRD patterns and Pawley fit of $Li(FSI)_{0.5}(TFSI)_{0.5}(GN)_2$.

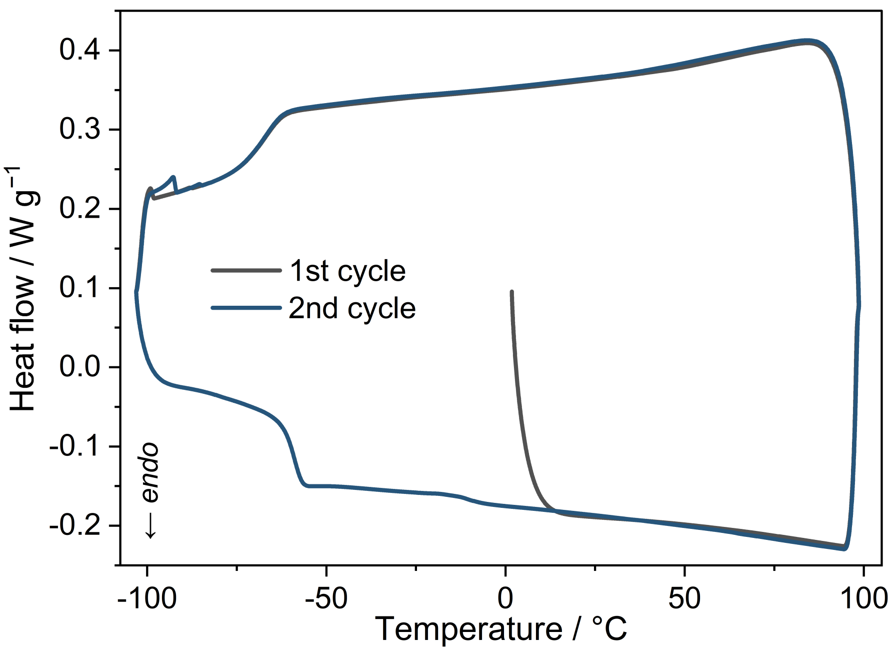

Figure S14. DSC curve of $Li(TFSI)(GN)_2$.

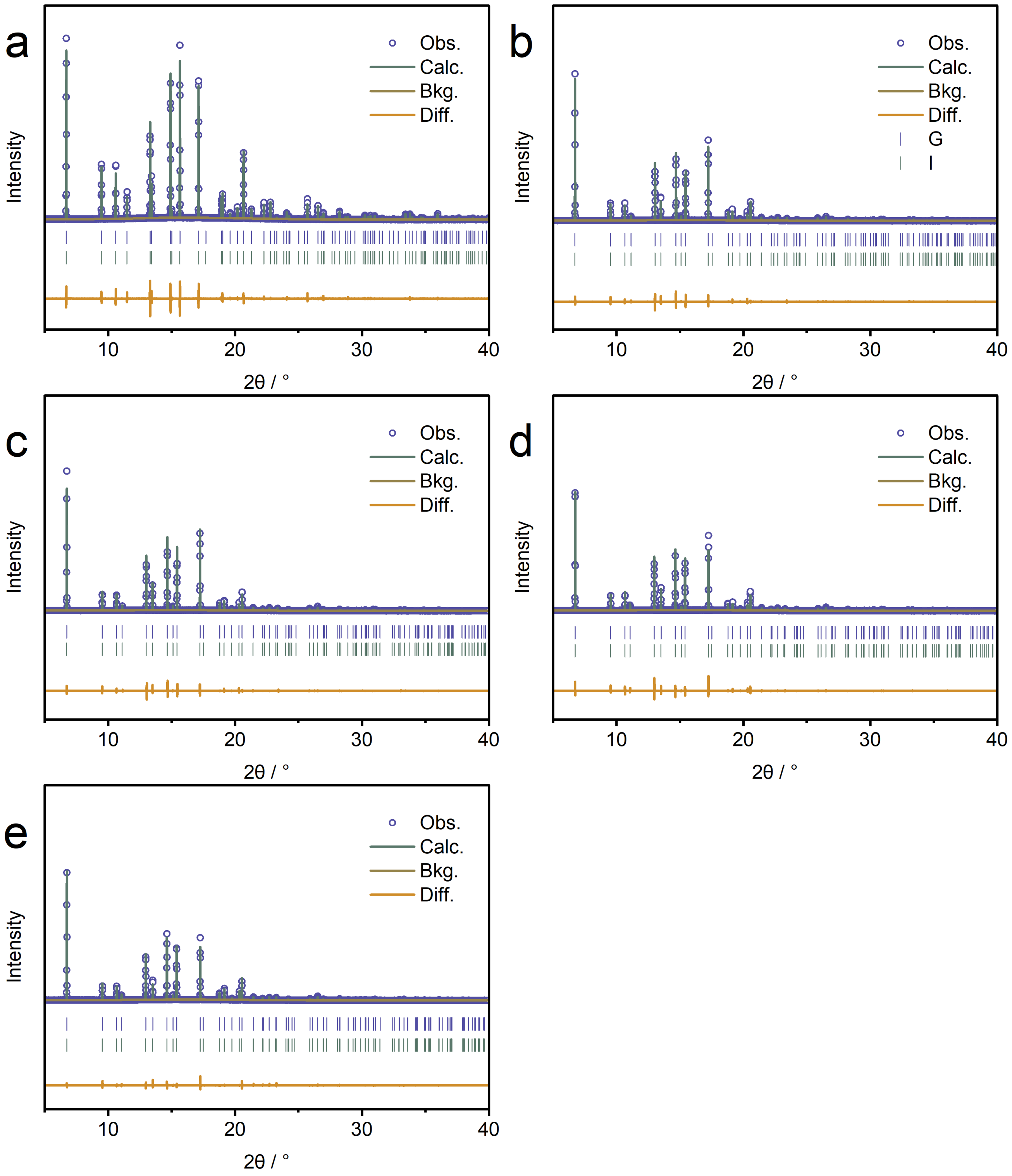

Figure S15. VT-PXRD patterns and Pawley fit of **1** at (a) −50, (b) 30, (c) 40, (d) 50, and (e) 55 °C.

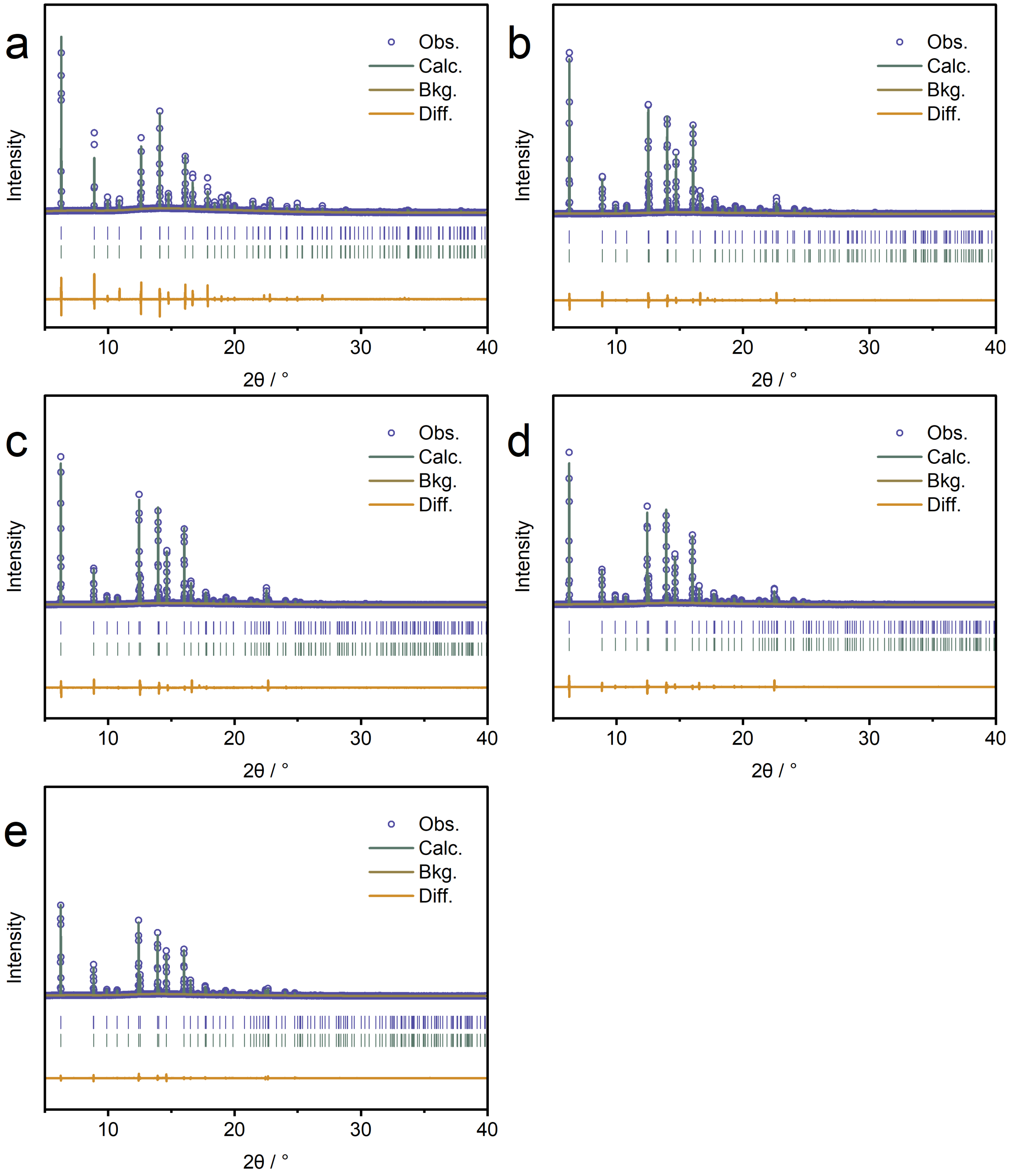

Figure S16. VT-PXRD patterns and Pawley fit of **2** at (a) 0, (b) 30, (c) 60, (d) 70, and (e) 80 °C.

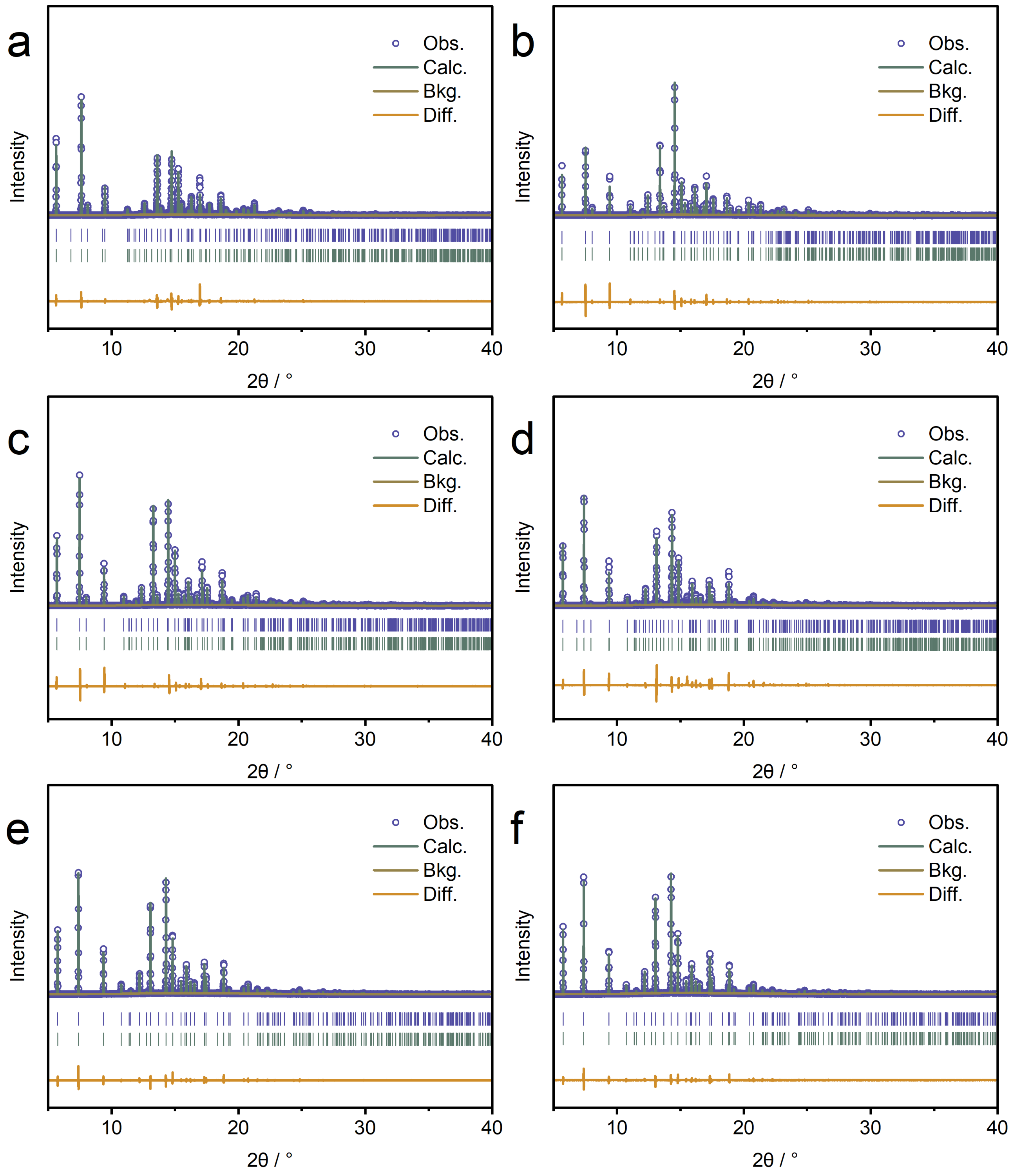

Figure S17. VT-PXRD patterns and Pawley fit of **3** at (a) −150, (b) 0, (c) 30, (d) 60, (e) 70, and (f) 80 °C.

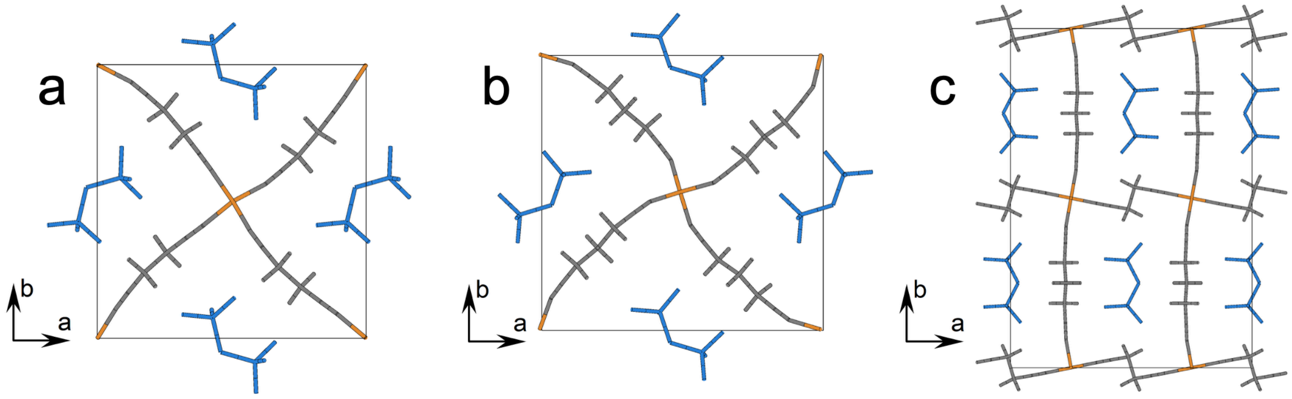

Figure S18. DFT-optimised geometry of (a) **1**, (b) **2**, and (c) **3** employed in the computational mechanical analyses.

a

$$\begin{pmatrix} 25.1 & 20.0 & 13.8 & 0.1 & -1.3 & -0.0 \\ 20.0 & 24.3 & 12.2 & 0.0 & -0.9 & -0.5 \\ 13.8 & 12.2 & 18.5 & -0.5 & -1.7 & -0.1 \\ 0.1 & 0.0 & -0.5 & 1.6 & 0.5 & -0.4 \\ -1.3 & -0.9 & -1.7 & 0.5 & 3.2 & 0.5 \\ -0.0 & -0.5 & -0.1 & -0.4 & 0.5 & 6.3 \end{pmatrix}$$

b

$$\begin{pmatrix} 13.0 & 9.4 & 5.7 & 0.7 & -0.7 & -0.4 \\ 9.4 & 10.9 & 4.7 & 0.6 & 0.2 & -0.6 \\ 5.7 & 4.7 & 12.2 & 0.3 & -0.1 & -0.7 \\ 0.7 & 0.6 & 0.3 & 1.7 & -0.5 & -0.1 \\ -0.7 & 0.2 & -0.1 & -0.5 & 2.2 & 0.0 \\ -0.4 & -0.6 & -0.7 & -0.1 & 0.0 & 5.5 \end{pmatrix}$$

c

$$\begin{pmatrix} 32.8 & 7.1 & 11.3 & -0.0 & -0.0 & -0.0 \\ 7.1 & 16.8 & 12.6 & -0.0 & -0.0 & -0.0 \\ 11.3 & 12.6 & 11.1 & -0.0 & -0.0 & -0.0 \\ -0.0 & -0.0 & -0.0 & 3.4 & -0.0 & -0.0 \\ -0.0 & -0.0 & -0.0 & -0.0 & 3.7 & -0.0 \\ -0.0 & -0.0 & -0.0 & -0.0 & -0.0 & 2.5 \end{pmatrix}$$

Figure S19. Elastic tensors of (a) **1**, (b) **2**, and (c) **3**, obtained in the computational mechanical analyses in the unit of GPa.

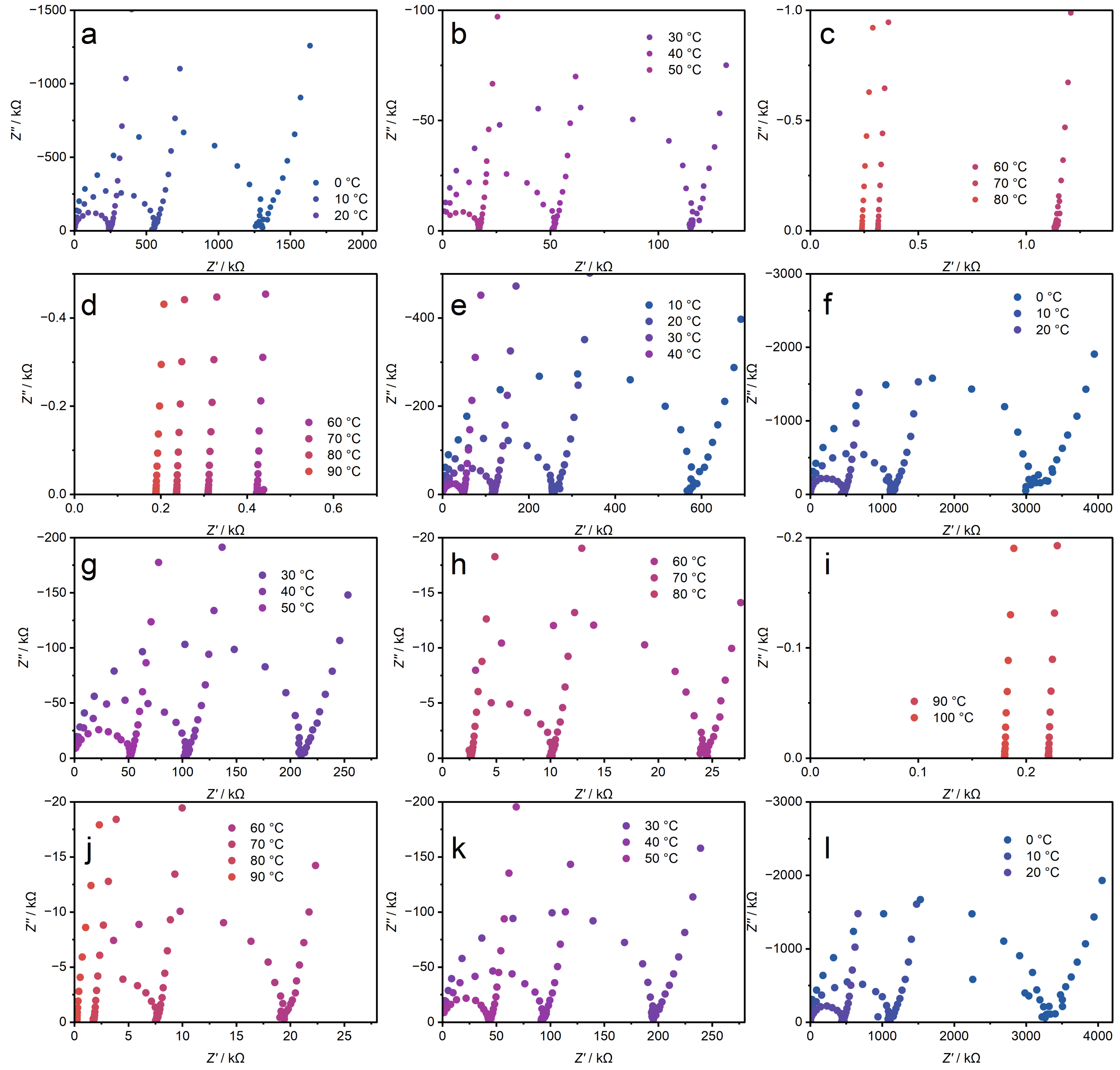

Figure S20. Nyquist plots of (a–c) heating process of **1**, (d, e) cooling process of **1**, (f–i) heating process of **2**, (j–l) cooling process of **2**.

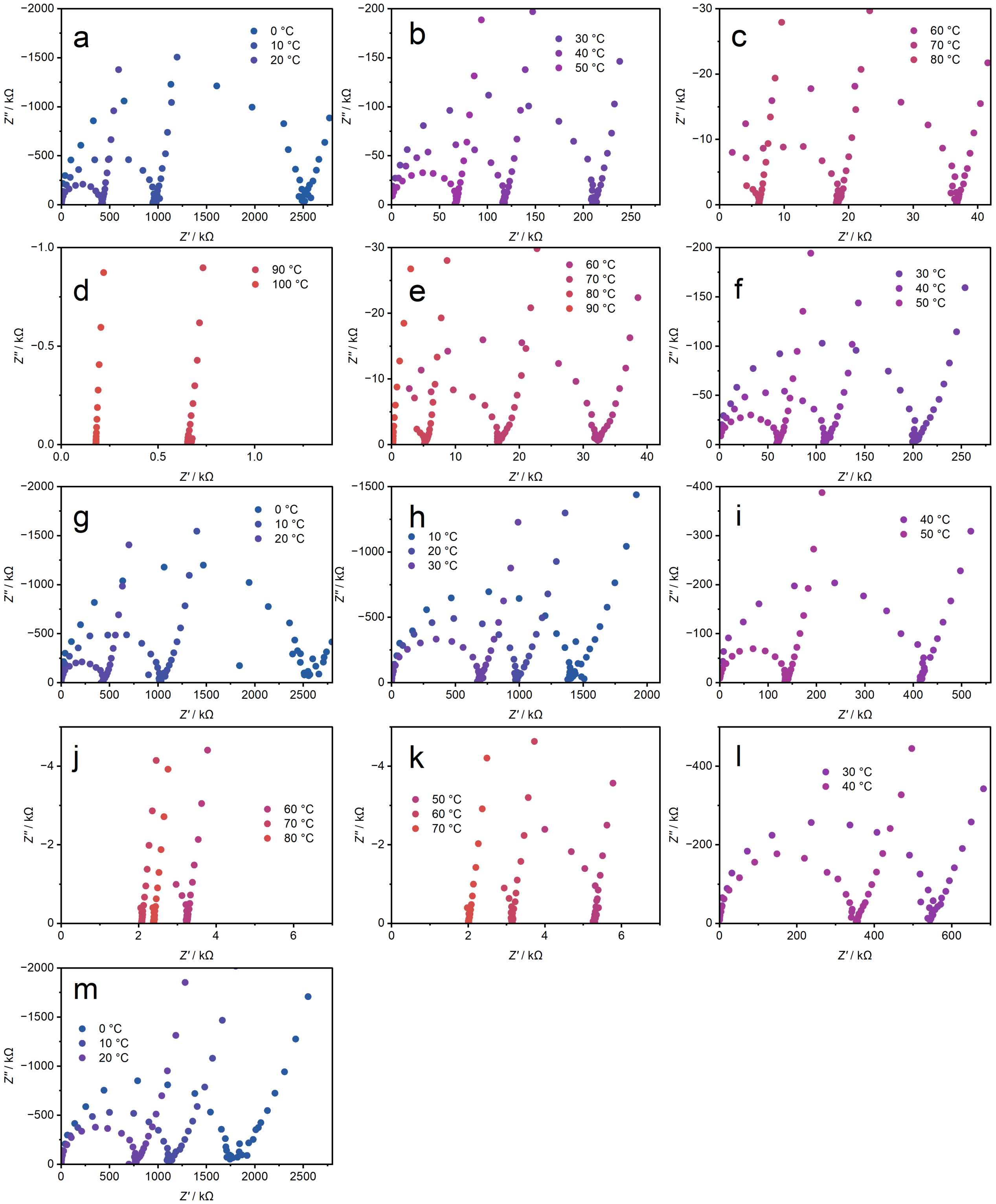

Figure S21. Nyquist plots of (a–d) heating process of **3**, (e–g) cooling process of **3**, (h–j) heating process of **4**, (k–m) cooling process of **4**.

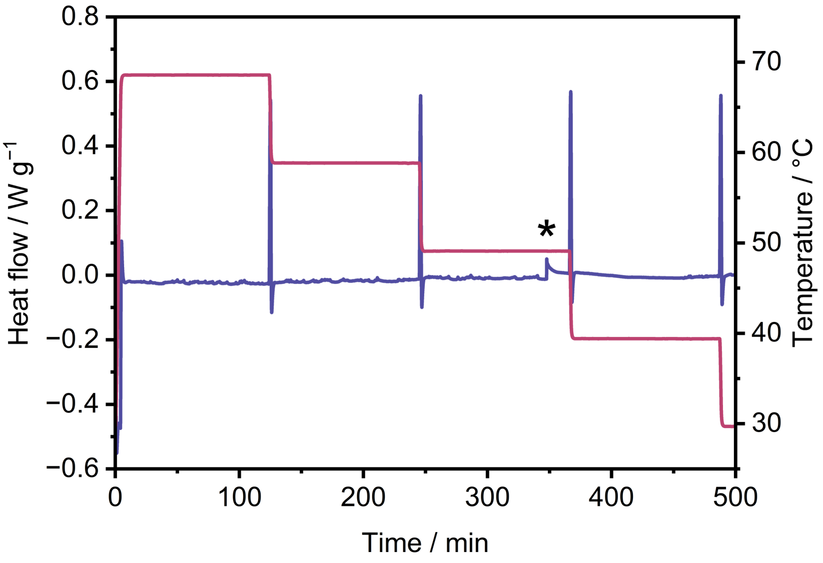

Figure S22. Temperature-step and isothermal DSC profile of **4**. The blue line represents the heat flow, and the red line represents the temperature program. Asterisk (*) indicates the crystallisation exotherm.

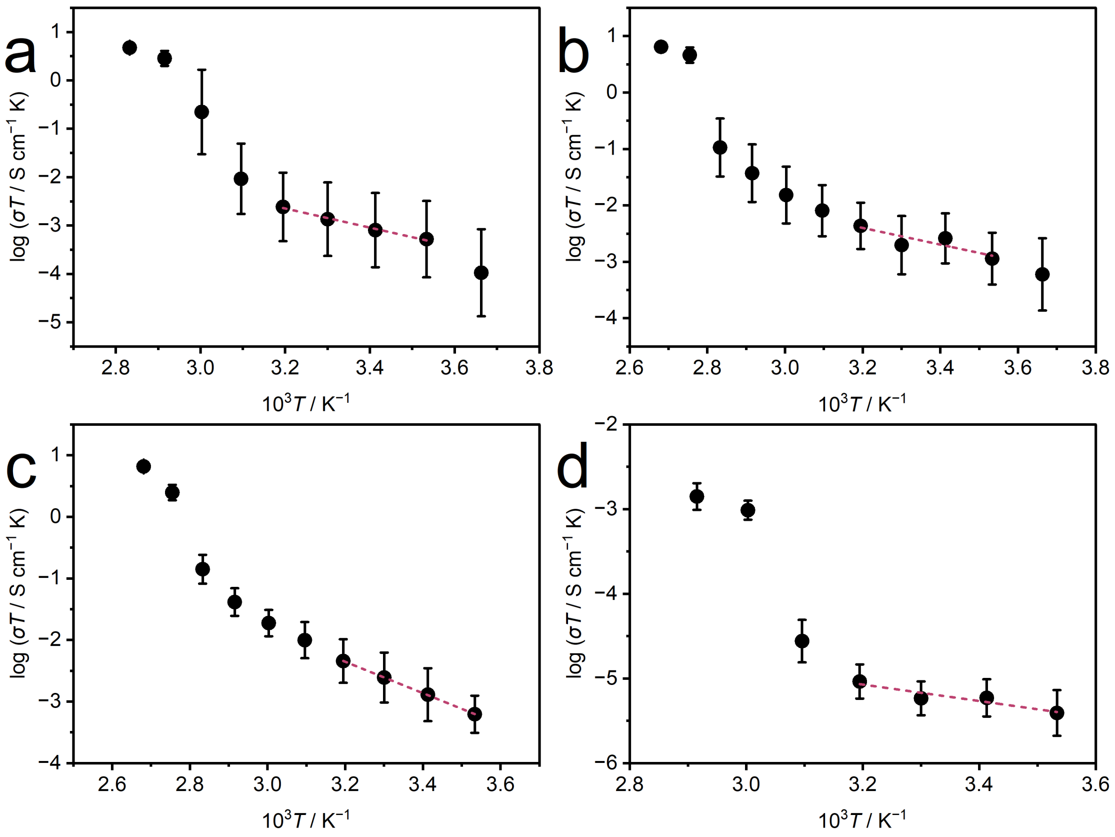

Figure S23. Arrhenius plots of conductivity with temperature-weighted conductivity of (a) **1**, (b) **2**, (c) **3**, and (d) **4** in the heating processes for determination of the activation energies. The dashed lines indicate the fitting results.

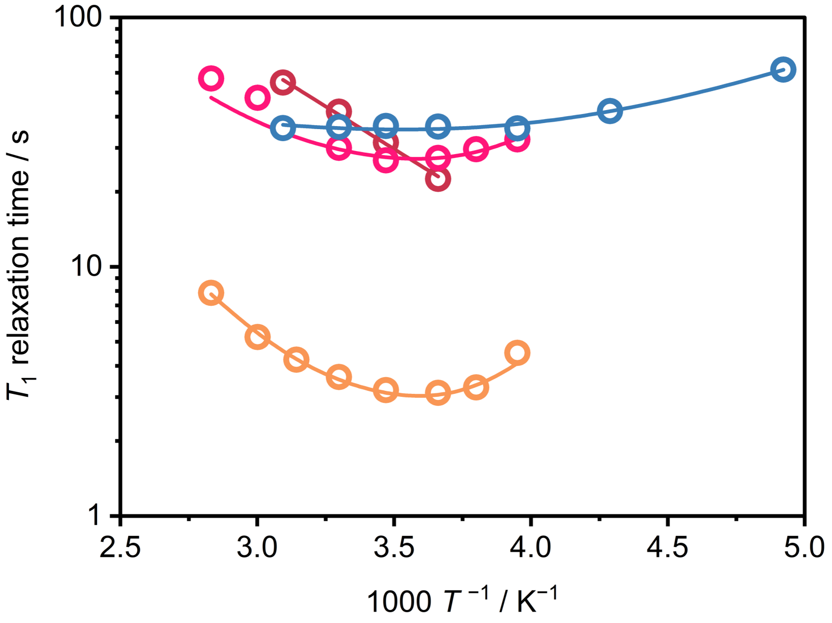

Figure S24. ^{7}Li relaxation time of **1** (red), **2** (yellow), **3** (pink), and **4** (blue). The solid lines indicate the fitting curves.

References

1 K. Tanaka, Y. Tago, M. Kondo, Y. Watanabe, K. Nishio, T. Hitosugi and M. Moriya, *Nano Lett.*, 2020, **20**, 8200–8204.

2 G. M. Sheldrick, *Acta Cryst.*, 2015, **A71**, 3–8.

3 G. M. Sheldrick, *Acta Cryst.*, 2015, **C71**, 3–8.

4 G. S. Pawley, *J. Appl. Crystallogr.*, 1981, **14**, 357–361.

5 N. Bloembergen, E. M. Purcell and R. V. Pound, *Phys. Rev.*, 1948, **73**, 679–712.

6 P. Giannozzi, S. Baroni, N. Bonini, M. Calandra, R. Car, C. Cavazzoni, D. Ceresoli, G. L. Chiarotti, M. Cococcioni, I. Dabo, A. Dal Corso, S. de Gironcoli, S. Fabris, G. Fratesi, R. Gebauer, U. Gerstmann, C. Gougoussis, A. Kokalj, M. Lazzeri, L. Martin-Samos, N. Marzari, F. Mauri, R. Mazzarello, S. Paolini, A. Pasquarello, L. Paulatto, C. Sbraccia, S. Scandolo, G. Sclauzero, A. P. Seitsonen, A. Smogunov, P. Umari and R. M. Wentzcovitch, *J. Phys. Condens. Matter*, 2009, **21**, 395502.

7 P. Giannozzi, O. Andreussi, T. Brumme, O. Bunau, M. Buongiorno Nardelli, M. Calandra, R. Car, C. Cavazzoni, D. Ceresoli, M. Cococcioni, N. Colonna, I. Carnimeo, A. Dal Corso, S. de Gironcoli, P. Delugas, R. A. DiStasio Jr, A. Ferretti, A. Floris, G. Fratesi, G. Fugallo, R. Gebauer, U. Gerstmann, F. Giustino, T. Gorni, J. Jia, M. Kawamura, H.-Y. Ko, A. Kokalj, E. Küçükbenli, M. Lazzeri, M. Marsili, N. Marzari, F. Mauri, N. L. Nguyen, H.-V. Nguyen, A. Otero-de-la-Roza, L. Paulatto, S. Poncé, D. Rocca, R. Sabatini, B. Santra, M. Schlipf, A. P. Seitsonen, A. Smogunov, I. Timrov, T. Thonhauser, P. Umari, N. Vast, X. Wu and S. Baroni, *J. Phys. Condens. Matter*, 2017, **29**, 465901.

8 R. Golesorkhtabar, P. Pavone, J. Spitaler, P. Puschnig and C. Draxl, *Comput. Phys. Commun.*, 2013, **184**, 1861–1873.

9 R. Gaillac, P. Pullumbi and F.-X. Coudert, *J. Phys. Condens. Matter*, 2016, **28**, 275201.

10 H. Katsuragawa, S. Mori, Y. Tago, S. Maeda, S. Matsuda, H. Toriu, R. Nakayama, S. Kobayashi, T. Hitosugi and M. Moriya, *ACS Appl. Energy Mater.*, 2025, **8**, 3599–3605.
